# Biomimetic Design of Biopolymer‐Based Water‐Resistant Food Packaging Materials

**DOI:** 10.1111/1541-4337.70653

**Published:** 2026-09-16

**Authors:** Ajahar Khan, Zohreh Riahi, Ruchir Priyadarshi, Jong‐Whan Rhim

**Affiliations:** ^1^ BioNanocomposite Research Center, Department of Food and Nutrition Kyung Hee University Seoul Republic of Korea; ^2^ Humanities Convergence Research Center, Department of Food and Nutrition Kyung Hee University Seoul Republic of Korea

**Keywords:** biodegradable packaging, bioinspired packaging design, biopolymer‐based, hydrophobicity, water resistance

## Abstract

With the growing demand for sustainable and eco‐friendly food packaging, biopolymer‐based materials are emerging as promising candidates due to their recyclability, biodegradability, and food safety. However, in practical applications dealing with high moisture content or perishable foods, their lack of water resistance poses a significant barrier, limiting their widespread use. This review comprehensively summarizes recent advances in biomimetic strategies to overcome these limitations of biopolymer‐based packaging, focusing specifically on water resistance and hydrophobic biopolymer‐based packaging systems. Inspired by natural surfaces, such as lotus leaves, mussel adhesive layers, insect cuticles, and nacre, researchers have developed functional films through nanocomposite reinforcement, hierarchical surface structuring, and chemical or biomimetic surface modification. It further provides an in‐depth discussion of fabrication techniques, structure–function relationships, and real‐world food applications, including fruits, vegetables, meat, seafood, bakery products, and liquid foods. It also highlights current challenges in scalability, biodegradability, and reproducibility, while introducing new tools, such as advanced characterization and machine learning, to accelerate innovation. Focusing specifically on water resistance, a crucial performance indicator, this review offers insights into developing robust, sustainable, and functional food packaging systems that can replace petroleum‐based plastics. Key findings suggest that nature‐inspired designs significantly improve water repellency without compromising biodegradability. Looking forward, bioinspired biopolymer systems hold strong potential to reshape future food packaging by aligning environmental safety with practical performance.

## 1 Introduction

Food packaging is a coordinated system for transporting, distributing, storing, and delivering food safely and healthily to consumers. It protects food from physical damage, contamination, and spoilage during storage and distribution. It also defends against environmental factors such as microbial contamination, moisture, oxygen, and light to ensure safety, maintain quality, and extend shelf life. To achieve these goals, four types of materials (paper, plastic, glass, and metal) are used alone or in combination. Among these, plastic is the most widely used because of its ease of processing into various shapes, excellent physicochemical properties, user‐friendliness, and cost‐effectiveness. When plastics were first industrialized in the 1950s, production was about 1.7 million tons. However, usage has grown exponentially, reaching 413.8 million tons annually in 2023 and projected to reach 590 million tons by 2050 (Anwar et al. 2025; Gibb 2019; Jaganmohan 2023; Plastics Europe 2015; Statista 2020). Plastics are used extensively across various industries, including packaging, construction, automobiles, electrical and electronics, and consumer goods. The widespread use of plastics in nearly every aspect of modern life has raised global concerns about long‐term environmental sustainability. Currently, the packaging industry consumes the most plastic, accounting for over 40% of total plastic production, with roughly half of that used for food packaging. Plastic materials are commonly used in applications that come into contact with food or drinks, especially in food packaging, kitchenware, cooking utensils, and food processing equipment.

However, plastics used in food packaging pose serious environmental issues because they can take hundreds of years to degrade naturally after being discarded. Since 1950, more than 8 billion tons of plastic have been produced globally, and about 80% of this plastic ends up as waste each year (Anwar et al. 2025). Furthermore, these plastics break down into tiny particles, including microplastics and nanoplastics, which can contaminate the environment and food. They also enter the human body through the food chain, potentially affecting human health and safety.

Rising concerns about the environmental impact of biodegradable plastic packaging, the depletion of natural resources, and food safety are driving demand for biodegradable packaging materials made from renewable resources as an alternative to synthetic plastics. These materials are especially popular for short‐term, single‐use applications, such as disposable tableware, plates, cups, kitchenware, diapers, trash bags, beverage containers, agricultural mulching films, fast‐food containers, and medical and personal protective equipment. Biopolymers are viewed as a promising, eco‐friendly alternative to traditional nonbiodegradable, nonrenewable plastic packaging. They offer advantages over synthetic plastics, including biodegradability, biocompatibility, and renewability, and some natural biopolymers are even edible (Edo et al. 2025). Additionally, biopolymer‐based materials, particularly when integrated with active agents such as antimicrobials or antioxidants, offer potential benefits for packaging, including the preservation of food quality and extension of shelf life by inhibiting microbial growth (Fang et al. 2025).

Furthermore, biopolymer films are well‐suited for incorporating various additives, such as antioxidants, antifungals, antibacterials, pigments, and other nutrients (Rostamabadi et al. 2024). Despite these advantages, several barriers hinder their commercialization, including high production costs, limited scalability, variability in raw material supply, and incompatibility with conventional plastic processing equipment (Panou and Karabagias 2023). Moreover, regulatory approvals for food contact materials and market acceptance remain ongoing challenges.

However, most biopolymers have relatively low mechanical and thermal stability, high hydrophilicity, and poor processability compared with plastics, which significantly limits their industrial uses (Panou and Karabagias 2023). To address these challenges in biopolymer‐based packaging materials, various methods have been used, including blending multiple polymers (W. Zhang et al. 2024), forming physicochemical crosslinks between or within polymer chains (W. Zhang et al. 2025), creating multilayer films (F. Xie 2023), coating the film surface with hydrophobic lipids or wax, and reinforcing polymers with nanomaterials to produce nanocomposite films (Sabu Mathew et al. 2025).

In particular, most biopolymer‐based packaging materials have low moisture resistance due to their hydrophilic nature, limiting their use for packaging foods with high moisture content, such as beverages, fruits and vegetables, meat, and fish. When moisture‐sensitive biopolymer‐based packaging materials absorb moisture in a high‐humidity environment, they may deform or lose strength, rendering them unable to perform their intended functions (Zhong et al. 2024). Additionally, moisture may condense on the surface of packaging film in some cases, affecting food quality or promoting the growth of microorganisms, thereby seriously compromising food safety. In some instances, packaging materials need more than basic moisture resistance: a superhydrophobic surface with a water contact angle (WCA) of 150° or higher (Zhong et al. 2025). Superhydrophobic packaging materials are being developed for food packaging to minimize residual food waste and contamination by offering superior water‐repellent and slip‐resistant coatings. These materials have been shown to reduce food residue and potential food loss up to 15% in some cases by stopping sticky foods like syrup, honey, and dairy products such as yogurt from sticking to the packaging walls but also help extend the shelf life of food by providing self‐cleaning, antimicrobial, and improved barrier properties (Li, Li, et al. 2025; Mohammadi et al. 2024; Ruzi et al. 2022; Zhang, Bi, et al. 2019).

Recently, efforts have focused on developing new packaging materials that mimic natural structures with specific functions, drawing inspiration from various biological forms (Dhatt et al. 2025; Fleetwood et al. 2026; Hu et al. 2025; G. Liu et al. 2024; Zhong et al. 2025). Biomimetic design, which replicates the unique hierarchical structure of natural materials, is an effective way to enhance the mechanical and moisture barrier properties of biopolymer‐based packaging materials. Bioinspired functional materials have been shown to significantly enhance performance compared to conventional biopolymer packaging in various aspects (Hu et al. 2025; G. Liu et al. 2024). Various biomimetic models inspired by natural phenomena are being used to create innovative food packaging materials. Specifically, models inspired by lotus leaves (Geng et al. 2023; Y. Liao et al. 2023), nacre (Liang et al. 2025), mussels (W. Zhang et al. 2025), and duck feathers (Wang, Jiang, et al. 2025) are well known for developing water‐resistant food packaging materials.

However, the literature clearly shows limitations in producing sustainable biopolymer‐based packaging materials that are water‐resistant and biocompatible for food packaging. Therefore, it is crucial to understand and confirm the full technology behind biomimetic water‐resistant packaging design when developing such materials. To clearly emphasize the novelty and relevance of this review article, it is essential to position it within the context of recent scholarly contributions. Zhong et al. (2025) provided a comprehensive overview of bioinspired and biomimetic strategies in food packaging, highlighting a range of functionalities such as mechanical reinforcement, barrier properties, and antimicrobial responsiveness (Zhong et al. 2025). However, their discussion lacked a focused evaluation of hydrophobic performance and water‐resistance mechanisms specifically in biopolymer‐based packaging systems. Similarly, Sabu Mathew et al. (2025) discussed hydrophobic modification of biopolymer composites, emphasizing various strategies for improving the water‐interactive characteristics of materials, but offered only limited insights into moisture management and long‐term stability under humid conditions. Hu et al. (2025) explored the broader landscape of hydrophobic packaging films, addressing synthetic and semi‐synthetic composites without narrowing the scope to biodegradable biopolymers and their biomimetic adaptations (Hu et al. 2025).

**FIGURE 1:**
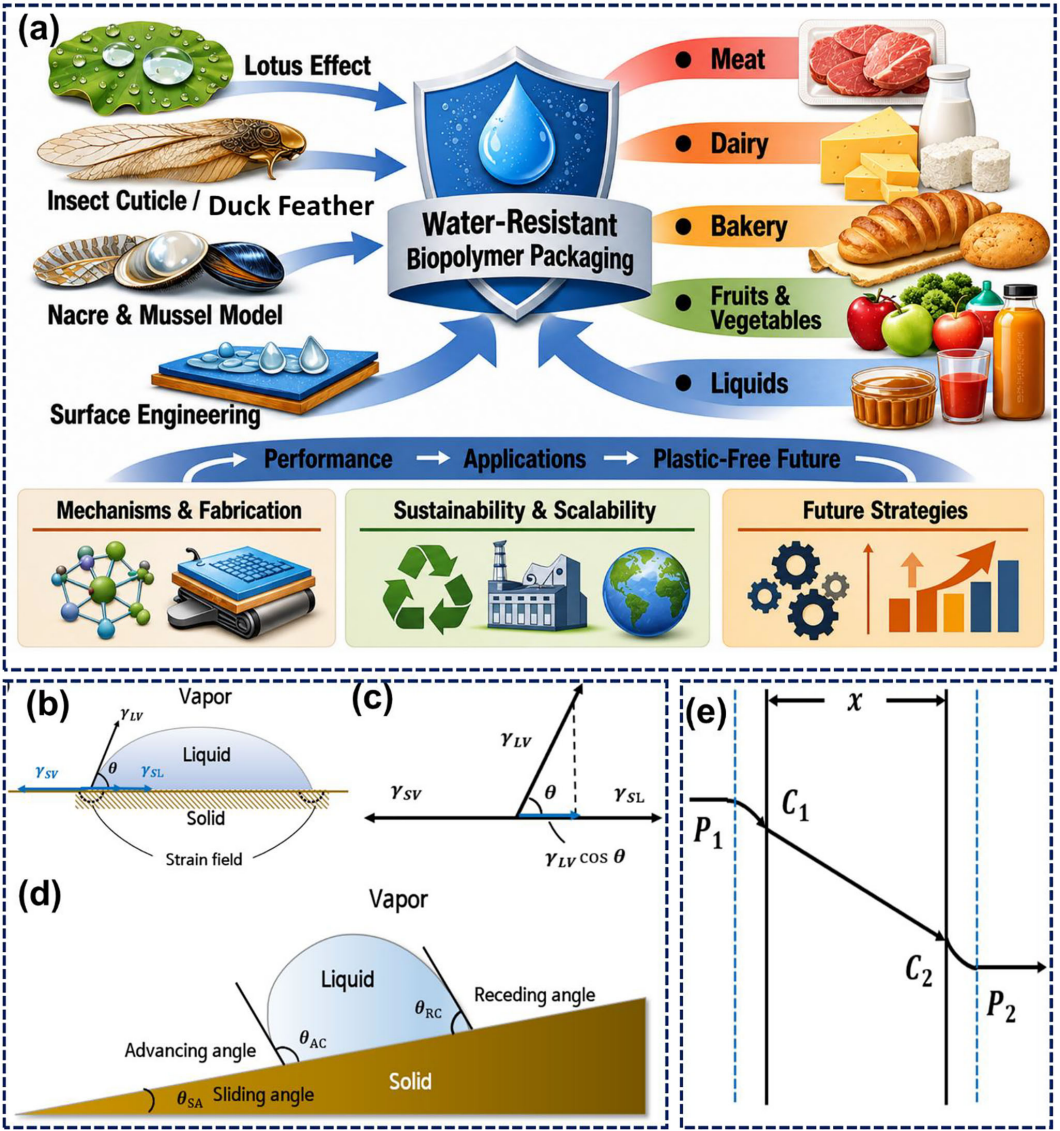
(a) Schematic of bioinspired strategies to enhance water resistance in biopolymer‐based packaging materials. Water contact angle on a flat film surface: (b) illustration of WCA and its relationship to the surface tension of the solid, liquid, and vapor phases. (c) Resolution of force vectors for the surface tension of a water drop on a solid surface. (d) Illustration of sliding angle and advancing and receding angles. (e) Water vapor permeation through a film in a steady state.

In contrast, this review emphasizes bioinspired design, particularly as applied to biopolymer‐based food packaging systems that aim to overcome inherent water sensitivity. It bridges a critical gap by systematically exploring both foundational mechanisms and advanced fabrication strategies, ranging from nanocomposite integration to surface engineering, that directly address the water resistance required for sustainable packaging systems. Furthermore, it classifies these developments by food type (meat, dairy, baked goods, fruits/vegetables, and liquids) and discusses scalability, sustainability, and future strategies (Figure 1a). As such, this review offers an integrative, application‐focused, and water‐resistance‐centered narrative that not only complements but significantly extends the current state of knowledge, providing practical direction for researchers and developers seeking to transition toward plastic‐free, high‐performance packaging alternatives.

## 2 Basics of Hydrophilicity and Hydrophobicity of Films

The wettability of a material, characterized by the contact between a liquid and a solid surface, is crucial in influencing fluid behavior on packing materials. Wetting is a universal phenomenon, yet its degree varies considerably on the basis of the chemical composition, surface energy, and morphology of the solid and liquid phases. Numerous entities in nature have developed surfaces with specific wetting properties, ranging from hydrophilic to hydrophobic, to adapt and thrive in various circumstances (Ahmad et al. 2018). The divergent behaviors of water on various surfaces are often characterized by the labels hydrophilic and hydrophobic. Hydrophilic surfaces strongly interact with water, leading to enhanced wetting, spreading, and adhesion. Conversely, hydrophobic surfaces resist wetting and promote droplet beading, whereas the ease of droplet roll‐off is further governed by dynamic wetting factors such as contact‐angle hysteresis and water adhesion (Law 2014). Hydro denotes “water,” philic indicates “affinity,” whereas phobic suggests “repulsion.” A surface is classified as hydrophobic when its static WCA (*θ*) exceeds 90°, and as hydrophilic when *θ* is below 90°. This threshold is fairly arbitrary, as a small movement of even a few degrees can transition a surface from one category to another. Although Teflon and fluoroalkyl monolayers are generally considered hydrophobic on the basis of their high static WCAs, they can exhibit substantial water adhesion, indicating that static WCA alone may not adequately describe water adhesion (Law 2014). This observation is relevant to packaging materials because surface wettability and liquid adhesion can affect moisture resistance, coating uniformity, printability, interlayer bonding, and the retention of liquid foods on package surfaces.

At the molecular level, wetting behavior arises from electrostatic interactions, van der Waals forces, and other intermolecular factors that govern whether water spreads or beads on a surface (Ahmad et al. 2018). Contemporary analytical instruments, including surface‐force equipment, atomic force microscopy (AFM), and molecular dynamics simulations, have elucidated these interactions and the associated surface energy profiles that govern wettability (Basinska 2017). Surface wettability can be intentionally adjusted through chemical modification. Hydrophilic surfaces are often created by incorporating highly hydrated functional groups via polymer or copolymer grafting. Examples include polyacrolein, phosphorylcholine, polyglycidol (PGL), poly(ethylene oxide) (PEO), poly(2‐hydroxyethyl methacrylate), *N*‐acryloylaminoethoxyethanol, and carboxybetaine acrylamide (Basinska 2017). These polymers incorporate polar moieties such as –(CH_2_)_2_OH, –CH(CH_2_OH)CH_2_O–, –CH_2_CH_2_O–, –OH, –CHO, and –CONH_2_, which enhance hydrogen‐bond formation and improve surface hydration (Basinska 2017). Conversely, hydrophobicity can be enhanced by attaching low‐surface‐energy chains or creating hierarchical surface roughness that entraps air pockets between the liquid and the solid, thereby reducing the effective contact area and inhibiting water infiltration. Modulating the hydrophilic–hydrophobic equilibrium through deliberate surface modification thus constitutes a pivotal strategy for engineering biopolymer films with customized wetting characteristics (Cui et al. 2023).

Grafting polymers can achieve controlled wettability by creating dense mono‐ or multilayers, applying hydrophobic copolymer films, or depositing nanoparticles to modify local surface chemistry and shape. In food packaging, high hydrophilicity is detrimental because water interactions compromise film integrity (Cui et al. 2023). Hydrophilic films lose mechanical strength upon moisture absorption, and in extreme cases, they may dissolve (Ye et al. 2020). Water molecules form hydrogen bonds with hydrophilic groups in biomacromolecules, resulting in swelling that weakens cohesion by diminishing cross‐linking density. Therefore, improving hydrophobicity is crucial to expand the functional capabilities of natural biopolymer films.

Hydrophobicity is quantified by the WCA, the angle formed between a water droplet and the surface of a film. Surfaces with a WCA greater than 90° are generally considered hydrophobic; however, many packaging studies categorize films with a WCA above 65° as moderately hydrophobic (Cui et al. 2023). Because static WCA does not fully represent water adhesion or droplet mobility, advancing and receding contact angles should be considered where these properties are relevant to the intended packaging application. Hydrophobicity can be enhanced by reducing surface free energy, increasing surface roughness, or combining both strategies. Therefore, understanding the balance between hydrophilicity and hydrophobicity is key to improving the performance of biopolymer films. By carefully modifying surface chemistry and structure, it becomes possible to control water interactions, leading to the development of waterproof, stable, and environmentally friendly packaging materials suitable for long‐term food preservation.

## 3 Water Resistance Properties of Packaging Films

The water resistance of a film is a crucial property for food packaging, especially for products with high moisture content, as it indicates the film's ability to withstand water and moisture. The water resistance of packaging films depends on several factors, including the chemical composition of the polymer matrix and additives or fillers (hydrophilic or hydrophobic), the chemical structure (such as polarity and cross‐linking), and the surface characteristics of the film. The wetting and water‐resistance properties of packaging films are commonly evaluated using metrics such as the WCA, water vapor transmission rate (WVTR) or water vapor permeability (WVP), water solubility (WS), and water absorption (WA) or swelling rate (SR).

### 3.1 Water Contact Angle

When moisture contacts the surface of food packaging film, the surface becomes wet, depending on the properties of both the moisture and the film. The wetting ability of the packaging film, the capacity of moisture to spread across its surface, is a key factor when selecting high‐moisture food packaging materials. The moisture wetting capacity of a packaging film surface is typically measured by assessing the WCA, which is the angle formed between the water droplet and the vapor interface (Figure 1b). More than 200 years ago, Thomas Young (1805) proposed treating the contact angle of a liquid as the result of the force balance of a resting on a plane solid surface under the action of three surface tensions (Thomas Young 1805). At equilibrium, the balance between the three surface tensions and the contact angle (Figure 1c) forms:

(1) $$\gamma_{\mathrm{SV}}-\gamma_{\mathrm{SL}}=\gamma_{\mathrm{LV}}\cos\theta$$

where θ is the WCA, *γ*_LV_, *γ*_SL_, and *γ*_SV_ are the surface tensions at the interfaces of the liquid and vapor phases, the solid and liquid phases, and the solid and vapor phases, respectively. The WCA is a useful indicator of the wettability and hydrophilicity or hydrophobicity of packaging films. A WCA of 0° indicates that a liquid completely wets a solid and spreads easily across the surface. A WCA greater than 0° indicates that the liquid does not spread. Typically, a film surface is considered hydrophilic if the WCA is below 90° and hydrophobic if it exceeds 90°. In particular, a surface is considered superhydrophilic when the WCA is under 10° and superhydrophobic when it exceeds 150° (Li, Li, et al. 2025; Mohammadi et al. 2024).

Other important parameters related to WCA measurement include the sliding angle (SA) of the water droplet, the advancing (θ_AC_) and receding (θ_RC_) WCAs (Figure 1d), and contact angle hysteresis (CAH), which is the difference between the advancing and receding WCA (i.e., θ_AC_–θ_RC_) (Ruzi et al. 2022). In general, CAH is used as an indicator of a water droplet's adhesion to a solid surface. That is, the higher the CAH, the greater the tendency of a water droplet to stick to the film surface (Miwa et al. 2000). The SA is not equal to the CAH, but film surfaces with a low SA often exhibit a low CAH. Materials with superhydrophobic surfaces are typically characterized by a WCA greater than 150° and a SA less than 10° (Ruzi et al. 2022). It is important to note that WCA, whether static, advancing, or receding, is a single‐point measurement typically obtained under ambient laboratory conditions immediately after film fabrication. It therefore does not necessarily predict performance after prolonged humidity exposure, repeated wetting‐drying cycles, or mechanical abrasion encountered during real packaging use, handling, and transport. Consequently, WCA retention after such stresses, rather than the initial WCA value alone, should be regarded as a more meaningful indicator of practical water‐resistance durability.

### 3.2 Water Vapor Permeability

When there is a significant difference in humidity between the inside and outside of the packaging, moisture can pass through the film walls, causing the food inside to either absorb moisture or dry out, which can affect its quality. The moisture permeability of packaging materials, known as WVP, is a unique property that depends on the material type. WVP indicates how readily water vapor passes through a packaging film, based on the vapor pressure difference across it. The higher the vapor permeability of a packaging film, the faster water vapor can pass through the packaging wall; therefore, WVP is used as a measure of the rate at which water vapor passes through the packaging wall.

As shown in Figure 1e, moisture movement through the film wall occurs in three stages. First, moisture is adsorbed and dissolved on the film wall surface at high moisture vapor pressure; then, diffusion toward the wall surface occurs at lower moisture vapor pressure; finally, it evaporates back into the air on the opposite wall, thereby causing moisture transfer. A combination of Fick's first law of diffusion and Henry's law of solubility is used to express the steady‐state permeability of water vapor through a packaging film. According to Fick's first law, the permeate flux, *J*, depends on water vapor diffusivity (*D*), the concentration difference (*dC*) in the film, and the differential of film thickness (*dx*). The following equation expresses the permeate flux:

(2) $$J=-D\frac{dC}{dx}$$

Henry's law states that the water vapor concentration (*C*) in the film is equal to the product of the solubility coefficient (*S*) and the water vapor partial pressure in the adjacent air (*P*):

(3) C=SP

Combining Equations (2) and (3) gives the following:

(4) $$J=-DS\frac{dP}{dx}$$

By rearrangement, the quantity *DS*, also known as permeability, can be expressed as

(5) $$DS=-J\frac{dx}{dP}=\mathrm{WVP}$$

Experimentally, WVP (g m/m^2^ s Pa) can be determined by the expression as follows:

(6) $$\begin{array}{ccc} \mathrm{WVP} & = & \frac{\mathrm{permeate}\;\mathrm{weight}\;\times \;\mathrm{film}\;\mathrm{thickness}}{\mathrm{surface}\;\mathrm{area}\times \mathrm{time}\;\times \;\mathrm{partial}\;\mathrm{pressure}\;\mathrm{difference}}\\ & & =\frac{\mathrm{WVTR}\;\times \;x}{\Delta P} \end{array}$$

where WVTR is the WVTR (g/m^2^ s) of the film, representing the amount of water vapor transmitted per unit area and time under specific test conditions and equal to *J* in Equation (4).

### 3.3 WA and WS

When biopolymer films come into contact with high‐humidity environments or moisture, moisture first adsorbs onto the film surface and then migrates internally through diffusion. During this process, the packaging film swells, reducing its strength or causing deformation of the packaging materials. As the amount of absorbed moisture increases, it acts as a solvent, dissolving the components of the packaging material and causing the material to lose functionality. Factors such as the film type, surface energy, fillers, and cross‐linking all influence the moisture resistance of biopolymer films. The WA rate and WS of a packaging material are key indicators of its ability to resist moisture.

The percentage of WA is calculated as follows:

(7) $$\mathrm{Water}\;\mathrm{absorption}\;\left(\%\right)=\frac{W_{f}-W_{i}}{W_{i}}\times 100$$

where the final and initial weights of the film sample are denoted by *W_f_* and *W_i_*, respectively.

Similarly, the WS of a film is determined by immersing a dry film in water for a specific time and measuring the mass loss due to dissolution. To determine the WS of a biopolymer film, first measure the initial dry mass (*M*_1_) of the film sample. Then, soak it in water for a specified period and dry it again until the mass remains constant (*M*_2_). Finally, calculate the WS of the film as follows:

(8) $$\mathrm{WS}\;\left(\%\right)=\frac{M_{1}-M_{2}}{M_{1}}\;\times 100$$

## 4 Bioinspired Nanocomposites

Bioinspired nanocomposites use nature's design principles to develop more durable packaging films with improved water resistance. Traditional bio‐based packaging materials often lack adequate mechanical strength and are highly moisture‐sensitive. Insights from natural systems, such as hierarchical nanoscale structures, can inform solutions to these issues, resulting in high‐performance bio‐based packaging that is resistant to water damage (Hu et al. 2025). Building on these ideas, researchers have developed bioinspired nanocomposites that markedly enhance the moisture barrier and durability of sustainable packaging films.

A primary technique involves integrating high‐aspect‐ratio nanofillers into a polymer matrix to replicate a “brick‐and‐mortar” architecture. Nanoclays are platelet‐like minerals (e.g., montmorillonite, halloysite, and bentonite) that disperse throughout biopolymers, forming convoluted pathways for water molecules (Perera et al. 2024). The nanoclay sheets function as impermeable “bricks” integrated into the polymer “mortar,” compelling water vapor to traverse a longer, zigzag pathway rather than a direct one (Calambas et al. 2021). This significantly reduces water transfer; research on starch‐based films indicates that adding a minimal amount of clay can decrease WVP and impede moisture absorption in the material. Moreover, polymer films augmented with montmorillonite show markedly reduced WVP and enhanced mechanical strength, as the uniformly dispersed clay layers form an efficient barrier and fortify the matrix (Perera et al. 2024). Nanoclay‐added composites exhibit enhanced moisture resistance and stiffness relative to the unmodified biopolymer, a phenomenon commonly noted in biodegradable packaging films. The use of nanoclays in diverse bio‐based matrices consistently enhances both the mechanical and barrier characteristics of the packaging while maintaining its compostability (Enescu et al. 2019). Despite these barrier benefits, the food contact safety of nanoclays depends on filler type, surface modification, and migration potential. Montmorillonite is generally regarded as low‐risk when used at low loadings (typically <5 wt%) with minimal nanoparticle release, whereas organically modified nanoclays containing quaternary ammonium surfactants require separate migration assessment, as the surfactant itself may migrate even if the clay platelets remain immobilized in the polymer matrix (Bumbudsanpharoke and Ko 2015). Migration testing following EU Regulation (EU) No. 10/2011 and compliance with specific migration limits are therefore necessary before nanoclay‐reinforced films can be used in direct food contact. Nanoclay‐based bio‐nanocomposites have been utilized in food packaging to enhance shelf life; for instance, clay‐reinforced starch or polylactic acid (PLA) films provide superior protection for sensitive foods against humidity and spoilage, surpassing traditional packaging films in preserving diverse food products (Pech‐Cohuo et al. 2024; Purbey et al. 2025). The efficacy of nanoclays illustrates the potential of bioinspired layering: by mimicking natural layered composites at the nanoscale, more robust and water‐resistant packaging materials can be obtained.

**FIGURE 2:**
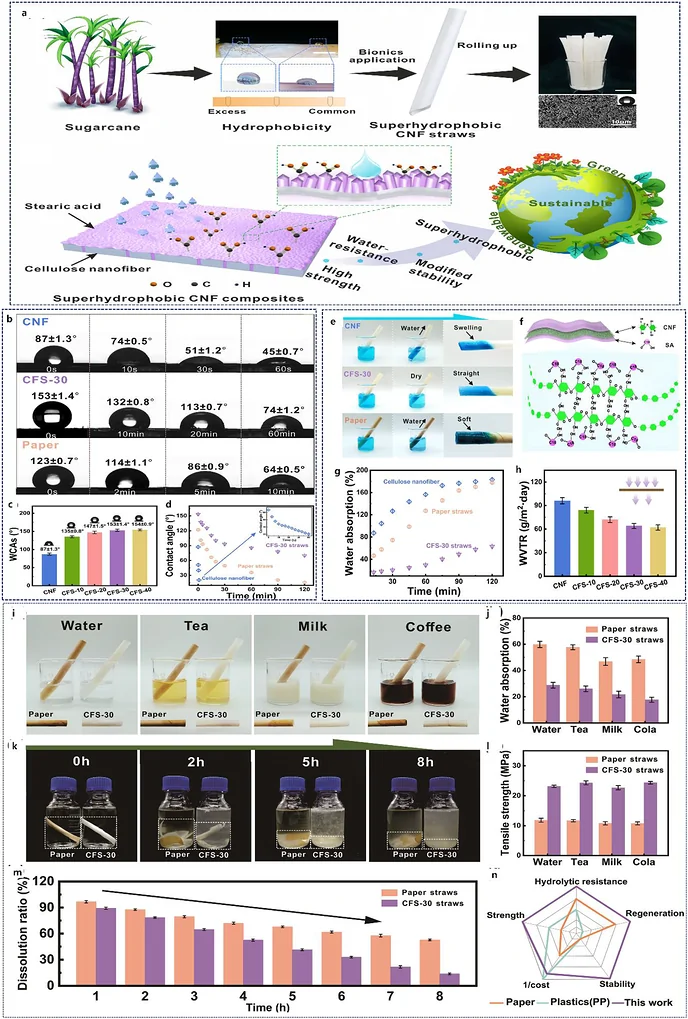
Schematic illustration of the bioinspired design and performance of superhydrophobic modified cellulose nanofiber straws (CFS) modeled after the hierarchical surface of sugarcane peel (a). The CFS straws exhibit high water contact angles (WCA) versus cellulose nanofiber (CNF) and paper straws (b–c) and maintain stable WCA over time (d). Water immersion and absorption tests (e–f) show low moisture uptake, attributed to the strong hydrogen‐bonding network (g). CFS also demonstrates reduced water vapor transmission rates (h). Practical tests confirm CFS‐30 retains structure after 30 min in beverages (i), with lower water absorption (j) and greater bending strength (k) than paper straws. Dissolution resistance after 8 h is shown (l and m), and a radar chart (n) highlights the superior hydrophobicity, strength, durability, and biodegradability of CFS compared to plastic and paper straws. Reprinted with permission from (Qin et al. 2022).

Cellulose nanocrystals (CNCs) and nanofibers, derived from the omnipresent structural polymer cellulose, are another category of bioinspired nanomaterials that enhance water resistance (K. Huang et al. 2023). CNCs are rod‐shaped crystalline nanoparticles derived from plant fibers; they exhibit exceptional strength and form percolating networks stabilized by hydrogen bonds. Cellulosic nanocrystals, when included in biopolymer films, improve barrier efficacy by occupying micro‐gaps and forming a thick, cohesive matrix that obstructs moisture and gas infiltration (de Lima et al. 2025). Cellulose‐based nano‐additives are derived from the structural composition of plant cell walls, where a matrix of polysaccharides is fortified with nanoscale cellulose fibrils, resulting in enhanced toughness and resistance to external stress. Likewise, including CNCs in packaging sheets can significantly reduce WVP and WA. In thermoplastic starch films, elevating the CNC content to around 20% significantly reduced WVP, suggesting that a critical network of nanocellulose substantially impedes water passage (Csiszár et al. 2021). The resultant composite exhibits enhanced dimensional stability in humid environments, attributable to the rigid, hydrogen‐bonded CNC network, while also acquiring increased strength and oxygen‐barrier qualities. Pure cellulose films, although exceptional oxygen barriers, are inherently hydrophilic and hence lack significant water resistance in isolation (Nasiri et al. 2024). To resolve this issue, researchers integrate CNCs with hydrophobic polymers or utilize surface coatings, resulting in hybrid films that exhibit enhanced moisture resistance. A technique involved the application of layer‐by‐layer (LBL) coatings of chitosan and CNC onto a PLA substrate, resulting in a transparent “green” barrier that markedly reduced water vapor transfer (Halász et al. 2015). Cellulose nanoparticles are now utilized in edible coatings and bioplastic packaging to enhance structural integrity and somewhat augment water resistance (Florido‐Moreno et al. 2025). Moreover, motivated by the hydrophobic properties of sugarcane peels, researchers engineered stearic acid (STA) (10, 20, 30, and 40 wt%, respectively) modified cellulose nanofiber straws (CFS). Among all compositions, CSF‐30 (cellulose nanofiber modified with 30 wt% of STA) exhibits superhydrophobicity (WCA ∼153°), substantial tensile strength (67.15 MPa), and remarkable recyclability (Figure 2a–d) (Qin et al. 2022). In comparison to commercial paper straws, CFS exhibited enhanced water resistance, structural integrity in diverse liquids, reduced WVTR, and expedited, cleaner biodegradation (Figure 2e–h).

CFS straws were tested against commercial paper straws in water (0°C and 65°C, 30 min) and in tea, milk, and coffee; CFS showed no swelling or bending (Figure 2i). Compared with paper straws, CFS exhibited ∼50% lower water uptake and ∼120% higher wet tensile strength (Figure 2j,l). In a 7 wt% NaOH/12 wt% urea freeze‐thaw (FT) dissolution test at −12°C (Figure 2k), after 8 h the remaining fragments were 52.87% ± 3.17% for paper straws versus 13.69% ± 2.87% for CFS (Figure 2m). Overall comparisons of hydrophobicity, regeneration, strength, stability, and cost among paper, plastic, and CFS straws are summarized in Figure 2n. Although performance is promising, the reported fabrication involves vacuum filtration, manual film rolling, immersion in STA/ethanol for 2 h, and drying at 120°C. These steps raise concerns about processing time, solvent recovery, coating uniformity, and energy demand in large‐scale operations. The estimated production cost was reported to be approximately 0.7 cents per CFS straw, compared with approximately 2 cents for PLA straws and 4 cents for commercial paper straws. However, these values were derived from laboratory‐scale life‐cycle cost assumptions; pilot‐scale or industrial‐scale techno‐economic analyses are needed to determine whether the reported cost advantage remains valid after accounting for solvent recycling, energy consumption, equipment investment, labor, quality assurance, and supply chain costs. Moreover, the “biodegradability” claim refers to dissolution in an NaOH/urea solution rather than real composting conditions, which may not reflect true end‐of‐life performance. Their renewability and biodegradability make them attractive for food packaging, providing bioinspired nanoscale reinforcement similar to wood fibers without harsh synthetic chemicals. Current work focuses on improving the moisture barrier of cellulose‐based nanocomposites (e.g., for fresh produce packaging and paper coatings) by optimizing CNC dispersion and/or grafting hydrophobic groups to achieve breathable yet water‐repellent films.

Emerging two‐dimensional (2D) nanomaterials offer a novel bioinspired approach to significantly improving barrier performance. An exemplary instance is MXene nanosheets (e.g., Ti_3_C_2_T_x_, a stratified metal carbide), which combine a high aspect ratio with functional surface chemistry (Khezerlou et al. 2025). Although synthetic, MXenes replicate the LBL structure observed in natural nacre‐like composites; they can be oriented parallel to the film surface to form a practically impermeable barrier against water and gases (Khezerlou et al. 2025). Recent investigations indicate that trace amounts of MXene in biodegradable films significantly reduce water permeability. Gelatin‐based nanocomposite films containing about 0.75% MXene exhibited significantly reduced WVP due to the in‐plane alignment of MXene sheets, which obstructs moisture transport (M. et al. 2025a). MXene enhances the film by augmenting tensile strength and Young's modulus, while also providing a more hydrophobic surface that reduces WA (W. Liu et al. 2023). In starch or polyvinyl alcohol (PVA) matrices, MXene fillers similarly establish a convoluted pathway and robust interfacial contacts, resulting in a decrease of almost 91.2% in WVP in certain studies (M. Dong et al. 2025b). Exceptional barrier performance positions MXene‐infused bio‐nanocomposites as viable choices for water‐resistant packaging. Moreover, MXenes can offer ancillary advantages pertinent to food packaging: their nanosheets frequently incorporate antimicrobial metal ions or can be functionalized with bioactive compounds, imparting antimicrobial efficacy against foodborne pathogens alongside moisture protection (Khezerlou et al. 2025). The multifunctionality of MXene‐based nanocomposites enables their use as active packaging materials that keep freshness by restricting moisture and oxygen intrusion, hence preventing spoiling.

Nevertheless, the safety of MXene‐containing materials for direct food contact requires careful evaluation. MXene toxicity may depend on its elemental composition, surface functional groups, particle size, concentration, oxidation state, and exposure duration (Lim et al. 2021; J. Wu et al. 2022). In a Ti_3_C_2_T_x_‐PLA film study, MXene migration was reported to be low; however, migration potential increased at higher MXene contents and may consequently increase toxicological risk (Santos et al. 2022). In contrast, no reduction in cell viability was observed for a specific MXene‐based nanocellulose film formulation in an in vitro cytotoxicity test (Tang et al. 2023). These findings indicate that safety should be assessed individually for each MXene type, synthesis route, film formulation, and intended food contact condition. Before commercial food packaging applications can be considered, migration studies should evaluate the release of MXene nanosheets, titanium‐containing oxidation products, fluorine‐containing surface residues, and other synthesis‐related constituents into appropriate food simulants. In addition, MXene‐based packaging must comply with applicable food‐contact regulations, which require that packaging materials do not transfer constituents to food at levels that may endanger consumer health or adversely affect food quality. Despite being a recent development in the bioinspired material category, preliminary studies suggest that MXenes may represent a “next frontier” in food packaging technology, owing to their superior barrier qualities against moisture, gases, and light (Khezerlou et al. 2025). However, comprehensive migration, toxicological, long‐term stability, and regulatory evaluations are still needed before MXene‐based films can be used commercially as direct food contact materials.

In practice, bioinspired nanocomposites, such as those mentioned, are enabling the development of water‐resistant, biodegradable packaging that was previously unattainable with pure biopolymers. By emulating nature's micro‐ and nano‐scale structural techniques, such as stacking platelets, entangling nanofibers, or aligning 2D sheets, scientists have developed films that repel water, safeguard contents, and preserve structural integrity in humid conditions. These improvements are now being implemented in food packaging applications. These advancements highlight bioinspired nanocomposites that integrate natural design principles with advanced nanotechnology, producing sustainable packaging materials that are water‐resistant without the use of petroleum‐based plastics. By employing the ideas summarized below, researchers are continuing to expand the approaches of bioinspired nanocomposites, advancing us toward packaging solutions that exhibit resilience and versatility inspired by nature's designs.

## 5 Bioinspired Hydrophobic Surface Models

Nature provides a vast array of surface patterns that demonstrate exceptional water resistance and other notable characteristics. Researchers have studied these biological systems to devise bioinspired approaches that enhance the water resistance, mechanical strength, and multifunctionality of biopolymer packaging materials. Prominent natural templates include superhydrophobic plant leaves, resilient protein‐based adhesives from marine species, water‐repellent animal textures, and durable multilayer composites. This section explores several key models, such as lotus leaves, mussel byssus, duck feathers, nacre, insect cuticles, and integrated composite strategies, and their influence on the development of water‐resistant biopolymer films for food packaging (Table 1).

### 5.1 Lotus Leaf Model

**FIGURE 3:**
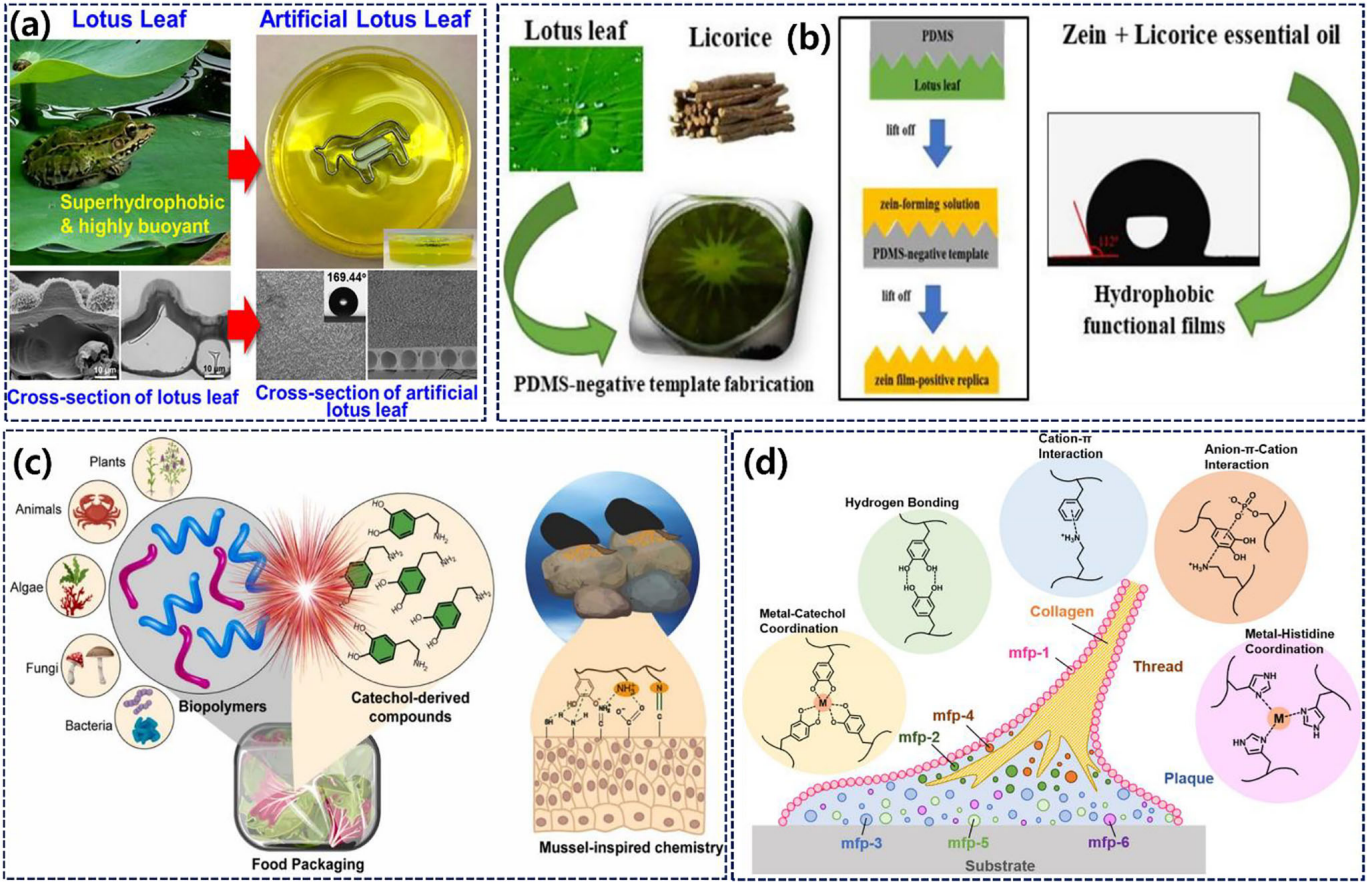
(a) Schematic illustration of a highly buoyant superhydrophobic composite mimicking a lotus leaf (Choi et al. 2014). (b) Lotus‐leaf‐inspired zein films using a PDMS mold and licorice essential oil to achieve micro/nano surface roughness and enhanced hydrophobicity (Luís et al. 2019). (c) Schematic illustration of mussel‐inspired chemistry towards fabricating biopolymer‐based water‐resistant packaging (W. Zhang et al. 2025). (d) The distribution of mussel foot proteins (mfps) in the byssus and their reversible interactions, including hydrogen bonding, electrostatic forces, π–π stacking, cation–π interactions, metal coordination, and covalent bonding (J. Chen and Zeng 2022). PDMS, polydimethylsiloxane.

Leaves of the lotus plant (*Nelumbo nucifera*) exemplify natural superhydrophobic surfaces. Their remarkable water repellency, sometimes referred to as the “lotus effect,” results from a combination of low‐surface‐energy waxes and a hierarchical micro/nanostructured surface topology (Luís et al. 2019). Water droplets form high contact angles (>150°) and slide off lotus leaves with minimal inclination, transporting dirt particles and facilitating self‐cleaning of the surface, even in muddy conditions (Figure 3a) (Choi et al. 2014). This extraordinary phenomenon results from multiscale surface roughness: Micron‐scale papillae on the leaf, each adorned with nanoscale wax protrusions, reduce the contact area and the adhesion of water.

Researchers have effectively replicated the lotus leaf's design to develop self‐cleaning hydrophobic films. A common approach involves replicating the dual‐scale roughness by molding or imprinting. Luís et al. (2019) developed a negative mold of a lotus leaf using PDMS (polydimethylsiloxane) and then cast zein (corn protein) films over it, thereby replicating the intricate pattern of the lotus leaf on the biopolymer surface. The zein film, particularly when enhanced with a low‐surface‐energy licorice essential oil, exhibited the distinctive lotus‐leaf micro/nano “rugosity” and achieved a high WCA of around 112.5°, indicating pronounced hydrophobicity (Figure 3b). In addition to their hydrophobicity, these bioinspired films are biodegradable and possess antibacterial and antioxidant properties derived from plant oil, making them viable sustainable alternatives to traditional plastic packaging. The lotus effect shows that mimicking a plant's waxy, textured surface significantly enhances a film's water resistance and self‐cleaning, which is advantageous for food packaging applications that require moisture barriers and anti‐fouling properties.

### 5.2 Mussel Model

Marine mussels firmly attach to damp rocks using byssal threads and adhesive plaques, inspiring water‐resistant adhesives for packaging materials (Fang et al. 2025). The byssus of mussels has exceptional strength, flexibility, and adhesive properties due to its distinctive chemical composition. Mussel foot proteins (mfps) contain a high concentration of 3,4‐dihydroxyphenylalanine (DOPA), a catechol‐modified amino acid primarily responsible for the strong interfacial interactions and cohesion of the byssus (Y. Li and Cao 2019). DOPA residues form several robust, reversible interactions with surfaces, including hydrogen bonds, covalent cross‐links formed through oxidative processes, metal‐ion coordination (e.g., with Fe^3+^), cation–π interactions, and π–π stacking with aromatic groups. These bonding mechanisms enable mussels to adhere to a range of surfaces underwater and to self‐repair. DOPA‐metal coordination complexes (e.g., DOPA‐Fe^3+^) in the byssal threads function as sacrificial cross‐links that fracture under stress and then rebuild, thereby dissipating energy and averting catastrophic failure. This mechanism imparts significant toughness and extensibility to the threads, enabling them to stretch over 30% before rupture while ensuring robust adhesion (Y. Li and Cao 2019).

Researchers have adapted mussel‐inspired chemistry for packaging by functionalizing biopolymers, such as proteins, chitosan, or cellulose, with catechol groups or by using mussel‐mimetic coatings, such as polydopamine, to enhance adhesion and water resistance (Figure 3c) (W. Zhang et al. 2025). Figure 3d shows the distribution of mfps within the byssus, highlighting their role in facilitating diverse reversible interactions, including hydrogen bonding, electrostatic forces, π–π stacking, cation–π interactions, metal coordination, and covalent bonding, that together ensure strong and resilient adhesion (J. Chen and Zeng 2022). In the mussel model, the catechol moieties of DOPA are available to connect with both inorganic fillers and organic matrices, thereby improving composite integrity. Coating cellulose‐based films with a thin layer of polydopamine can facilitate interfacial hydrogen bonding and cross‐linking, thereby decreasing water permeability and enhancing mechanical strength (W. Zhang et al. 2025). Furthermore, mussel‐inspired additives such as tannic acid (a polyphenol exhibiting catechol activity) have been included in biopolymer films to operate as natural cross‐linkers. These catechol‐containing additives establish robust networks of hydrogen bonds and metal coordination within the polymer, emulating the chemistry of mussel adhesive plaques and markedly enhancing the film's strength (Pandey et al. 2020). Tannic acid or dopamine modifications in biopolymer packaging films have been extensively studied, resulting in significantly enhanced barrier characteristics, tensile strength, and required functions, including antioxidant activity and UV resistance (W. Zhang et al. 2025). The mussel model exemplifies the application of bioinspired adhesive chemistry in creating packaging materials that are moisture‐resistant, even under damp environments, and possess robust integrity due to many reversible bonding interactions.

### 5.3 Duck Feather Model

Bird feathers, especially those of waterfowl, offer another example of the development of hydrophobic packing surfaces. Feathers from ducks, geese, or swans exhibit inherent superhydrophobicity owing to their microtextured structure and hydrophobic preen oils. The feather surface has a hierarchical structure: barbs and barbules (delicate hair‐like extensions from the quill) provide a rough, fiber‐like surface that entraps air, whereas these fibers are coated with waxy oils released by the bird to resist water (Wang, Jiang, et al. 2025). This structure promotes the beading and roll‐off of water droplets while providing a self‐replenishing mechanism. If the coating is disrupted, oil from neighboring barbules can redistribute over the affected area, helping maintain long‐term water repellency. Motivated by this, researchers have created biomimetic films that integrate micro‐patterned textures with hydrophobic coatings to replicate feather functionality.

**FIGURE 4:**
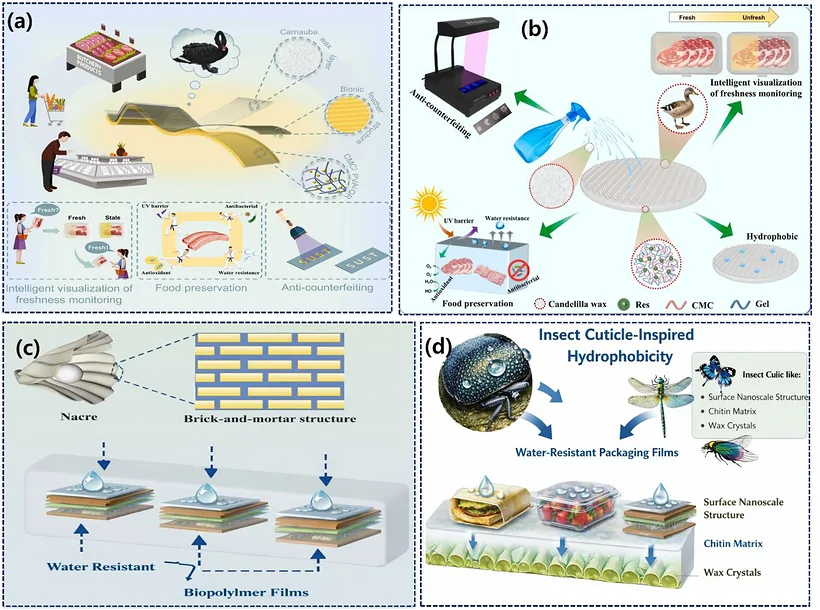
Schematic representations of biomimetic packaging concepts: (a) functional design and applications of bionic swan feather‐based carboxymethyl cellulose (CMC), polyvinyl alcohol (PVA), and quercetin (QR)‐based multifunctional films (Jiang et al. 2024); (b) structure–function overview of duck feather‐inspired CMC, gelatin (Gel), and resveratrol (Res)‐based packaging film (Wang, Jiang, et al. 2025); (c) conceptual model of nacre‐inspired water‐resistant biopolymer packaging; and (d) bioinspired design based on insect cuticle structures.

Jiang et al. (2024) developed a selective imitation of goose and swan feather surfaces for packaging films. A biopolymer film composed of carboxymethyl cellulose (CMC) and PVA was micro‐molded to emulate the barbed, anisotropic structure of feather surfaces (Figure 4a) (Jiang et al. 2024). Subsequently, they applied a plant wax (carnauba or candelilla wax) to the microstructured film to replicate the preen oil coating. The resulting composite film, designated C‐CPQ, exhibited markedly enhanced water resistance. Its feather‐like microstructure and wax coating increased the WCA while reducing the WVTR, thereby effectively limiting moisture penetration. The C‐CPQ film exhibited significant real‐world performance enhancements; specifically, beef encased in this film remained fresh for an additional 2 days compared to standard polyethylene (PE) wrap under the same conditions (Jiang et al. 2024). The bioinspired‐feather film exhibited hydrophobic characteristics while incorporating intelligent capabilities, including pH‐sensitive and luminescent components for freshness monitoring and tampering detection, all without sacrificing its barrier features. Similarly, another study developed a CMC/gelatin film inspired by duck feathers, with an asymmetric “barbed plumage” impression on one side and a candelilla wax coating applied via spraying (Figure 4b) (Wang, Jiang, et al. 2025). This asymmetric configuration offered one superhydrophobic surface to impede water and another more hydrophilic surface to permit limited breathability. The film successfully maintained the dryness of packed beef and prolonged its refrigerated shelf life to around 5 days, compared to 3 days with conventional plastic, by inhibiting moisture buildup (Wang, Jiang, et al. 2025). These examples highlight the potential of the duck feather model: by integrating micro‐scale texture (to generate air pockets and roughness) with hydrophobic coatings, packaging films can attain superhydrophobicity similar to feathers, resulting in enhanced water barrier efficacy and food preservation (Table 1).

**TABLE 1:** Conceptual comparison of bioinspired models for water‐resistant food packaging materials.

Parameter	Lotus‐leaf model	Mussel model	Feather model	Nacre model	Insect cuticle model	Composite model
Core mechanism	Hierarchical micro/nano surface roughness combined with low‐surface energy wax reduces solid–liquid contact area	Catechol (DOPA)‐mediated reversible interfacial bonding (H‐bonds, metal coordination, π–π stacking) provides wet adhesion and cross‐linking	Fibrous barb/barbule roughness entraps air, combined with a self‐replenishing oily coating for water repellency	Rigid platelet “brick” and soft biopolymer “mortar” arranged in staggered lamellae to deflect cracks and lengthen diffusion paths	Multiscale wax‐covered ridges/nanopillars on cuticle provide anisotropic wetting and mechanical antimicrobial action	Sacrificial, reversible bonds (H‐bonds, hydrogen‐bonded β‐sheet‐like clusters) dissipate energy and enable self‐healing or high toughness
Typical WCA range	High (generally >130°), superhydrophobic self‐cleaning behavior is the defining trait	Not primarily a wettability strategy; WCA is usually moderate, as the goal is strong adhesion	High (superhydrophobic on one or both faces); can be engineered asymmetrically for one hydrophobic and one breathable side	Moderate and matrix‐dependent; WCA is a secondary outcome, not the design target	High (typically 130°–160°), often with directional/anisotropic wetting behavior	Variable; hydrophobicity is a secondary effect of hydrophobic domains rather than a primary design goal
Water vapor permeability (WVP)	Can lower WVP through surface roughness and wax layers, but barrier improvement is secondary to surface repellency	Coating cross‐linking (e.g., catechol/polyphenol chemistry) narrows polymer network free volume, reducing WVP moderately	Combined microtexture and wax coating can meaningfully reduce WVP, particularly in asymmetric designs balancing barrier and breathability	Generally the strongest WVP reduction among all models, since tortuous lamellar diffusion pathways directly target vapor/gas transport	Textured, wax‐coated cuticle‐inspired surfaces reduce WVP moderately, mainly via reduced surface wetting rather than diffusion path tortuosity	Indirect and inconsistent; WVP reduction depends on the specific hydrophobic domain content rather than being a primary function
Mechanical performance	Minimal enhancement; the model is not designed for load‐bearing or toughness improvement	Moderate to good improvement in tensile strength and extensibility via reversible sacrificial bonding, self‐healing potential	Minimal to moderate; mechanical role is secondary to barrier and freshness preservation function	Strongest mechanical enhancement; the layered rigid‐soft architecture markedly improves toughness and crack resistance over the base polymer	Limited quantitative mechanical enhancement; contribution is mainly structural/antimicrobial rather than load bearing	High; sacrificial‐bond networks and hierarchical crystalline domains can yield tensile strength and toughness exceeding many commodity plastics
Scalability	Moderate; lab‐scale templating is established, but continuous large‐area replication of fine dual‐scale roughness remains challenging	Relatively high; coating‐based application (dip, spray) is compatible with existing industrial coating lines	Moderate; requires combined micro‐molding and coating steps, adding process complexity vs. single‐step coatings	Low to moderate; layer‐by‐layer assembly, freeze‐casting, or self‐assembly methods offer precision but are slow and difficult to scale industrially	Low; nanoimprinting and direct biotemplating have limited scalability due to high precision requirements and low throughput	Low; multi‐component, multistep chemistries are largely confined to lab‐scale demonstration
Food contact safety	Generally favorable when using food‐grade waxes/biopolymers, but residual mold or texturing agents require migration testing	Favorable in principle, as catechol‐based cross‐linkers are naturally derived, but comprehensive migration/toxicology data are still limited across the field	Favorable; feather‐inspired coatings typically rely on already‐approved food contact waxes and common biopolymer matrices	Requires careful evaluation; inorganic nanofillers used as the “brick” component raise nanoparticle migration and toxicity considerations	Favorable for chitin/chitosan‐based systems given established food contact status; synthetic wing‐replica surfaces need independent validation	Variable; individual components are often food‐derived, but multifunctional systems generally lack comprehensive migration assessment
Cost implications	Low to moderate; base materials are inexpensive, but precision templating/imprinting adds processing cost	Low to moderate; natural cross‐linkers and simple coating equipment keep costs close to conventional coated packaging	Low; relies on inexpensive, already industrially used natural waxes	Moderate to high; nanofillers increase raw material cost, with cost scaling with filler sophistication	High; specialized templating/tooling and low throughput drive up unit cost	High; multistep chemistries and limited industrial precedent make this the costliest model overall
Biodegradability	High; typically fully biodegradable biopolymer‐wax systems	High; biopolymer matrices remain biodegradable, and catechol‐based cross‐linkers are naturally derived	High; common film matrices and natural waxes are inherently biodegradable	Variable; biodegradability depends on filler type, as inorganic/carbon‐based fillers can persist and slow overall degradation	High; chitin/chitosan systems are biodegradable, whereas synthetic replicas depend on the biodegradability of the base polymer	Moderate to high; generally biodegradable, although dense cross‐linking may slow degradation compared with unmodified biopolymers

Abbreviation: WCA, water contact angle.

### 5.4 Nacre Model

Nacre, or mother‐of‐pearl, the inner layer of mollusk shells, is a quintessential natural composite that demonstrates how layered structures can provide remarkable strength and moisture resistance. Nacre consists of more than 95% aragonite calcium carbonate platelets, rigid and brittle “bricks” arranged in a staggered lamellar configuration and bonded by a thin layer (about 5% by volume) of soft biopolymers, including chitin polysaccharide and proteins, serving as the “mortar” (Figure 4c) (Morsali et al. 2020). Each aragonite platelet is about 5–8 µm in diameter and 200–900 nm thick, but the organic layer interspersed between the platelets is only ∼10–50 nm thick (W. Chen et al. 2025). This ultrastructured “brick‐and‐mortar” architecture endows nacre with an exceptional balance of hardness, stiffness, and toughness. Pure aragonite has poor fracture toughness (∼0.25 MPa m^1/2^), whereas nacre exhibits a toughness of around 10 MPa m^1/2^, representing an increase of over 40 times, despite the predominance of mineral composition (Morsali et al. 2020). Nacre can absorb roughly 2000 times more energy before fracture than the ceramic alone. Toughening mechanisms include crack deflection and branching at multiple mineral–polymer contacts, widespread microcracking, platelet pull‐out, and the presence of “sacrificial bonds” within the organic matrix that fracture before mineral failure, thereby dissipating energy (Barthelat and Zhu 2011). The organic interlayers, although present in small proportions, are essential: They restrict and redirect fractures at the interfaces and provide limited slippage between platelets, thereby averting catastrophic crack propagation. Nanoscale asperities and mineral bridges between platelets enhance load transmission and bolster the structural integrity of nacre (Barthelat and Zhu 2011).

The architecture of nacre has inspired the development of multilayer bio‐nanocomposites for packaging that replicate its alternating soft and hard structure. Researchers can produce films that are both robust and resilient, as well as effective barriers to gases and water vapor, by incorporating high‐aspect‐ratio nanofillers (the “brick”) into a biopolymer matrix (the “mortar”). Clay nanosheets, graphene oxide, or chitin nanoplates can be oriented within polymer films to create a brick‐and‐mortar configuration akin to nacre (Madhav et al. 2023). Nacre‐like films exhibit significantly enhanced mechanical properties, even when composed of an inherently weak polymer matrix. Their layered architecture impedes crack propagation by forcing cracks to follow a tortuous path through the layers, thereby dissipating energy and preventing direct fracture (Xie, Zhang, et al. 2024). They also establish an extensive, labyrinthine diffusion pathway for water or gas molecules, yielding enhanced barrier efficacy, a sought‐after characteristic for food packaging to prevent moisture and oxygen ingress. Various manufacturing approaches have been used, including LBL deposition, solution casting with evaporation‐induced self‐assembly, and freeze‐casting to produce aligned lamellae (Gao et al. 2020). For example, bioinspired nanocomposite films incorporating montmorillonite clay or nacre‐mimetic graphene frameworks exhibit markedly superior tensile strength and modulus compared to the base biopolymer, concurrently decreasing WVTRs by an order of magnitude or more (H. Zhao et al. 2018). The food contact safety of graphene‐based fillers (graphene oxide, reduced graphene oxide) remains an active area of investigation. Several studies have reported low cytotoxicity of graphene‐based materials at low concentrations; however, higher concentrations of graphene oxide may induce oxidative stress. Moreover, graphene‐based nanomaterials currently lack explicit regulatory authorization from the European Food Safety Authority (EFSA) for direct food contact applications (Cebadero‐Domínguez et al. 2022; Liao et al. 2011). Their use in nacre‐inspired packaging should therefore be regarded as a promising but presently unapproved strategy requiring dedicated migration and toxicological validation.

Overall, the nacre model demonstrates that the hierarchical arrangement of rigid and pliable elements can provide packaging materials characterized by superior toughness (impact resistance) and reduced permeability, addressing two key limitations of many biodegradable films.

### 5.5 Insect Cuticle Model

The cuticular surfaces of several insects, such as their wings and eggshells, serve as sources of inspiration for hydrophobic, self‐cleaning, and antibacterial packaging surfaces (Figure 4d) (Zheng et al. 2025). Insect wings frequently exhibit intricate hierarchical micro‐ and nanostructures that confer exceptional water repellency. An exemplary case is the butterfly wing, which has overlapping scale‐like microstructures adorned with nano‐ridges and waxes, resulting in one of the most complex three‐dimensional periodic surface patterns found in nature (Zheng et al. 2025). The multiscale features, including microscale grooves on wing scales, secondary nanoscale ridges and cross‐bridges, and tertiary nanostripes, provide a superhydrophobic surface with contact angles typically between 130° and 160° (Han et al. 2017). Butterfly wings exhibit directional (anisotropic) wetting owing to the oriented, flexible nanotips on their surface scales. This asymmetric structure allows water droplets to move readily toward the wing edge while resisting motion in the opposite direction toward the body. This unidirectional self‐cleaning system ensures that rainwater or dew efficiently cleans the wing by transporting material outward, a feature that might inspire self‐cleaning packaging films that expel water and pollutants in a regulated manner (Hancock et al. 2012).

Other insects provide additional functional surface models. Dragonfly and cicada wings are not only superhydrophobic but also feature nanopillar arrays that physically disrupt germs upon contact, endowing them with inherent antibacterial properties. Engineers have begun replicating such surfaces to develop packaging sheets that resist microbial contamination. A common production method is topographic replication, in which researchers use techniques such as nanoimprint lithography or templating with actual insect wings to transfer micro/nanopatterns onto polymer films, mimicking the structure of lotus leaves (Hancock et al. 2012). Wan et al. (2021) used a dragonfly wing mold to cast a biopolymer film, employing a plant extract that solidified into the wing's nanopattern, resulting in a textured surface that markedly reduced the film's wettability and WVP compared with a smooth film (Wan et al. 2021). Likewise, soft lithography, photolithography, and hot embossing have been used to imprint rice leaf and butterfly wing designs onto PDMS, resulting in surfaces that replicate the low‐adhesion, self‐cleaning properties of the wings (Bixler and Bhushan 2014). Although these techniques may be technically challenging, the resulting biomimetic surfaces have significant advantages: water beads and rolls off, carrying dirt and bacteria away, thereby maintaining the package's dryness and cleanliness.

In addition to surface texture, insects also drive the development of useful material compositions. An interesting example is the “lac blanket” mimetic film created by Ma et al. (2024) for packing fresh vegetables. The lac bug (*Kerria lacca*) excretes a resinous shellac substance for self‐protection; its natural coating is hydrophobic and forms a permeable barrier. Researchers developed a biodegradable film by incorporating shellac‐derived hydrogel particles into a chitosan matrix, which controls gas exchange like an insect's permeable covering (J. Ma et al. 2024). The film's permeable structure functions as adjustable gas “valves,” regulating O_2_ and CO_2_ permeability to reduce the respiration rate of packed cut vegetables. This bioinspired design preserves food freshness more effectively than an unaltered film by inhibiting excess moisture and altering the internal environment of the packaging (J. Ma et al. 2024). Similarly, textures inspired by cicada wings are being investigated for edible coatings to impart antibacterial and moisture‐resistant characteristics, as the nanopillars can mechanically destroy bacterial cells while simultaneously repelling water (Román‐Kustas et al. 2020). The novel applications of insect cuticle models demonstrate a significant principle: incorporating micro/nanostructures and biomolecules from insects can produce packaging materials that are both water‐resistant and effective in maintaining food quality, either via antimicrobial properties or atmospheric regulation.

### 5.6 Composite Model

In many cases, the most effective bioinspired designs combine multiple natural principles to achieve a balance of properties, for example, a material that is simultaneously tough, hydrophobic, and self‐healing (Bixler and Bhushan 2014). The “composite model” encompasses tactics that integrate bio‐inspiration or develop novel functionalities by harnessing dynamic chemistries. Two significant concepts in this domain are derived from spider silk and self‐healing polymer networks.

Spider dragline silk is widely recognized as one of the most resilient materials, exceeding the energy‐absorbing capacity of most synthetic fibers before fracture (Kiseleva et al. 2020). The key lies in a distinctive molecular architecture: silk threads consist of proteins that form nanocrystalline β‐sheet domains (abundant in hydrogen‐bonded antiparallel β‐strands) within a semi‐amorphous matrix of glycine‐rich chains (Chan et al. 2020). The β‐sheet nanocrystals act as rigid, load‐bearing nodes, whereas the surrounding matrix is flexible and can expand under stress. Multiple sacrificial hydrogen bonds within and between the β‐sheet domains break and reform cooperatively as the fiber elongates, giving spider silk an exceptional combination of high tensile strength (about 1 GPa, comparable to steel) and elongation at break (20%–30%) (Chan et al. 2020). This yields approximately threefold greater toughness than in synthetic polymers such as Kevlar (Kiseleva et al. 2020). Researchers have focused on incorporating β‐sheet‐like crosslinks and a hierarchical network structure into biopolymers to replicate spider silk in packaging sheets. For instance, by integrating small protein segments or polyphenols that form structured hydrogen‐bonded clusters, one might establish a network of reversible linkages analogous to the crystalline regions of spider silk. Chang et al. (2023) demonstrated this concept by cross‐linking soy protein with a tannic acid‐derived network under controlled drying conditions. This process promoted the formation of densely hydrogen‐bonded β‐sheet aggregates, yielding a film with a tensile strength of approximately 100 MPa and toughness exceeding that of many commodity plastics (Chang et al. 2023). Zhang, Liu, et al. (2019) replicated the hierarchical self‐assembly of spider silk by incorporating lignosulfonic acid (a natural polyaromatic nanoparticle) into a PVA matrix. The lignin particles functioned as multifunctional crosslinkers, establishing a sacrificial bond network at the nanoscale, analogous to β‐crystals in silk. The PVA/lignin composite film exhibited a tensile strength of around 98.2 MPa and an elongation of around 282%, resulting in an exceptionally high toughness of approximately 172 J/g (Zhang, Liu, et al. 2019). These biomimetic nanocomposites derive their efficacy from a process inspired by spider silk. Under stress, multiple weak bonds sequentially break to facilitate deformation and energy dissipation, whereas their subsequent reformation enables the repair of microdamage. The result is a packaging film that is both water‐resistant, due to the hydrophobic properties of β‐sheets and lignin domains, and resilient against punctures, drops, and tears, surpassing the performance of conventional films.

Another aspect of the composite model is the development of materials that can self‐repair or adapt responsively to environmental changes, drawing inspiration from biological systems that frequently exhibit healing capabilities. A strategy employed in bio‐based packaging integrates dynamic noncovalent bonds that can dissociate and reform, inspired by the reversible crosslinks seen in spider silk and mussel adhesive. Smirnov et al. (2021) developed self‐healing chitosan films by plasticizing chitosan with a natural deep eutectic solvent (NADES) made of citric acid and choline chloride. This NADES both plasticizes and crosslinks the polymer chains through a dense network of hydrogen bonds and ionic interactions (Smirnov et al. 2021). If the film is severed or fractured, the many reversible connections at the interface may re‐form, essentially “adhesively” rejoining the material. Their chitosan‐NADES film regained over 72% of its initial tensile strength and over 56% of its elongation after fracture, simply by compressing the fragments together, indicative of a distinct self‐healing property. This feature is essential in packaging, as slight punctures or tears can self‐seal, thereby averting package failure and food spoilage. Furthermore, multiple bioinspired features can be integrated within a single composite design. For example, a film may combine a lotus‐inspired superhydrophobic surface with a self‐healing underlying layer or incorporate antimicrobial agents into a nacre‐inspired layered architecture based on insect‐cuticle analogues. These intricate designs pose challenges yet promise multipurpose packaging materials that are waterproof, mechanically robust, and intelligent in functionality. As research in bioinspired packaging advances, the composite model, which merges chemical and structural inspirations from diverse natural systems, embodies the cutting edge for developing high‐performance sustainable packaging that competes with or exceeds petroleum‐based plastics in various aspects.

### 5.7 Relationship Between Biological Inspiration, Structure, and Packaging Performance

**FIGURE 5:**
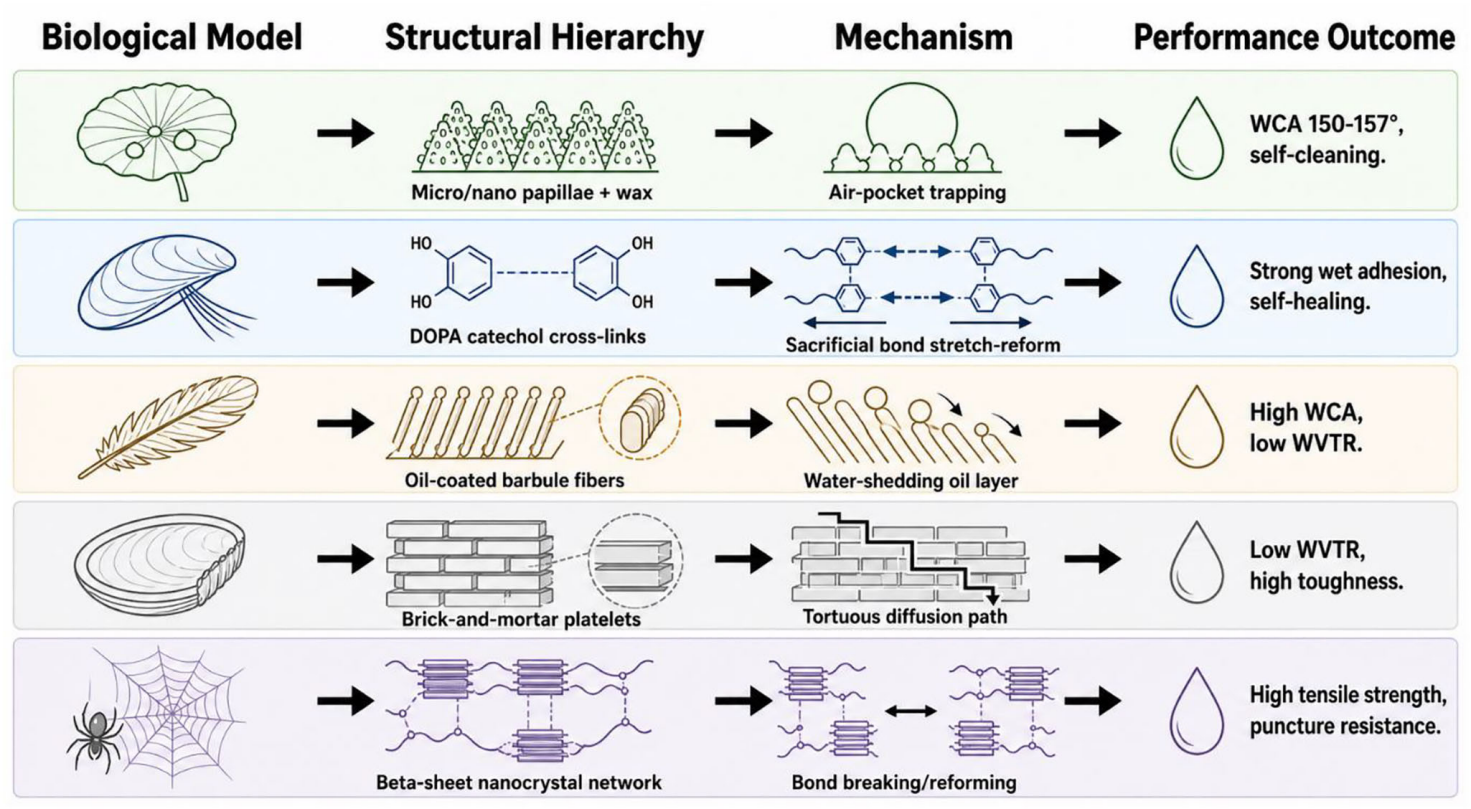
Biomimetic framework linking natural structures and water‐resistance mechanisms to hydrophobicity, barrier properties, and packaging performance. DOPA; 3,4‐dihydroxyphenylalanine; WCA, water contact angle; WVTR; water vapor transmission rate.

Although Sections 5.1–5.6 discuss each biomimetic model separately, they follow a common four‐step relationship. First, a biological system provides a natural model for controlling water. Second, this model is represented by a specific structural hierarchy, such as micro/nanoscale roughness, brick‐and‐mortar layers, or reversible molecular cross‐links. Third, these structures create a particular water‐resistance mechanism, including air trapping, tortuous diffusion pathways, or energy dissipation through sacrificial bonds. Finally, these mechanisms lead to measurable changes in hydrophobicity, barrier, and mechanical properties, such as WCA, WVTR, and tensile strength, which ultimately determine packaging performance, including shelf‐life extension, anti‐adhesion, and mechanical durability. For example, the dual‐scale papillae and waxy surface of lotus leaves create an air‐cushioned water‐repellent state, producing WCA values of >150° and strong self‐cleaning and anti‐adhesion behavior in liquid food packaging (Y. Li et al. 2018). Similarly, the brick‐and‐mortar structure of nacre forces water and gas molecules to follow longer, tortuous pathways, reducing WVTR while improving toughness (H. Zhao et al. 2018; Barthelat and Zhu 2011). In mussel‐ and spider silk‐inspired systems, reversible and sacrificial bonding improves self‐healing, puncture resistance, and water‐resistant performance (Y. Li and Cao 2019; Kiseleva et al. 2020). This integrated relationship (Figure 5) shows that hydrophobicity and barrier performance arise directly from the replicated biological structure and mechanism, providing a clear basis for selecting biomimetic strategies according to the intended packaging function.

## 6 Preparation of Polymer Nanocomposites

**FIGURE 6:**
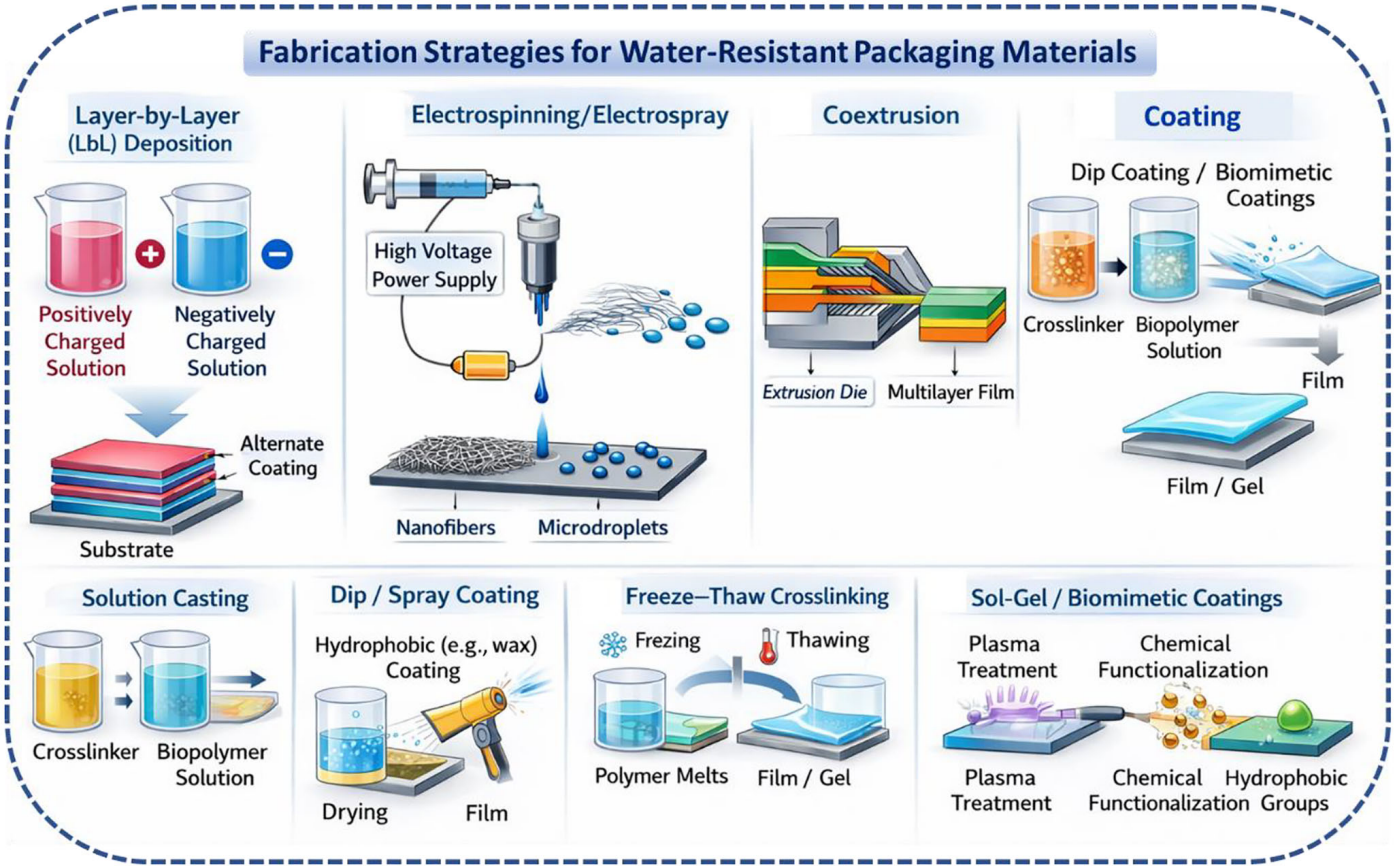
Schematic illustration of common methods used to develop water‐resistant food packaging materials.

Manufacturing techniques are crucial in determining the microstructure and water resistance of biopolymer‐based packaging films. Researchers have used a variety of methods, ranging from conventional solvent casting to advanced LBL assembly, electrospinning, FT cross‐linking, and sol–gel deposition, to fabricate bioinspired nanocomposites with enhanced hydrophobicity (Figure 6) (Xu et al. 2024). In addition, surface modifications, such as chemical grafting or biomimetic coatings, can also provide the desired hydrophobic characteristics required for packaging purposes. The preparation method affects film morphology (such as smooth or porous, layered or crosslinked) and therefore affects attributes such as WA, moisture barrier, and wettability. This section outlines the techniques and their impacts on the resulting water‐resistant packaging materials, highlighting meticulous process design that may replicate bioinspired models and mitigate the intrinsic hydrophilicity of biopolymer‐based packaging systems.

### 6.1 Solution Casting

This is the most straightforward and widely used technique for laboratory‐scale production of edible and biodegradable films. The biopolymer, together with any nano‐reinforcements, is dissolved or dispersed in a volatile solvent, cast onto a flat surface, and dried to evaporate the solvent, resulting in a uniform film (Xu et al. 2024). Casting is beneficial for generating homogeneous films of tunable thickness and for evenly incorporating fillers or active additives (Kang et al. 2021). Solution‐cast films often exhibit a dense morphology, enhancing their gas barrier properties; nevertheless, the intrinsically hydrophilic characteristics of biopolymers frequently result in elevated WVP and moisture sensitivity (Khan et al. 2024). To address these constraints, researchers have adopted bioinspired methodologies using in situ cross‐linking and nanomaterial reinforcement to improve the water resistance and performance of solution‐cast films (Xu et al. 2024). For instance, Tang et al. (2023) utilized a nacre‐mimetic approach, employing a green solution‐casting and solvent‐evaporation technique with 1D TEMPO‐oxidized nanocellulose (TNF) and 2D MXene to create an interwoven stack structure, incorporating 0D AgNPs to occupy the voids (Tang et al. 2023). This bioinspired assembly produced a robust interface and dense structure, granting the TNF/MX/AgNPs film characteristics that are significantly superior to those of PE films, along with enhanced acid–base stability. The film demonstrated ultra‐low oxygen permeability, validated by molecular dynamics simulations, and exhibited superior barrier characteristics against volatile organic vapors compared to PE films.

**FIGURE 7:**
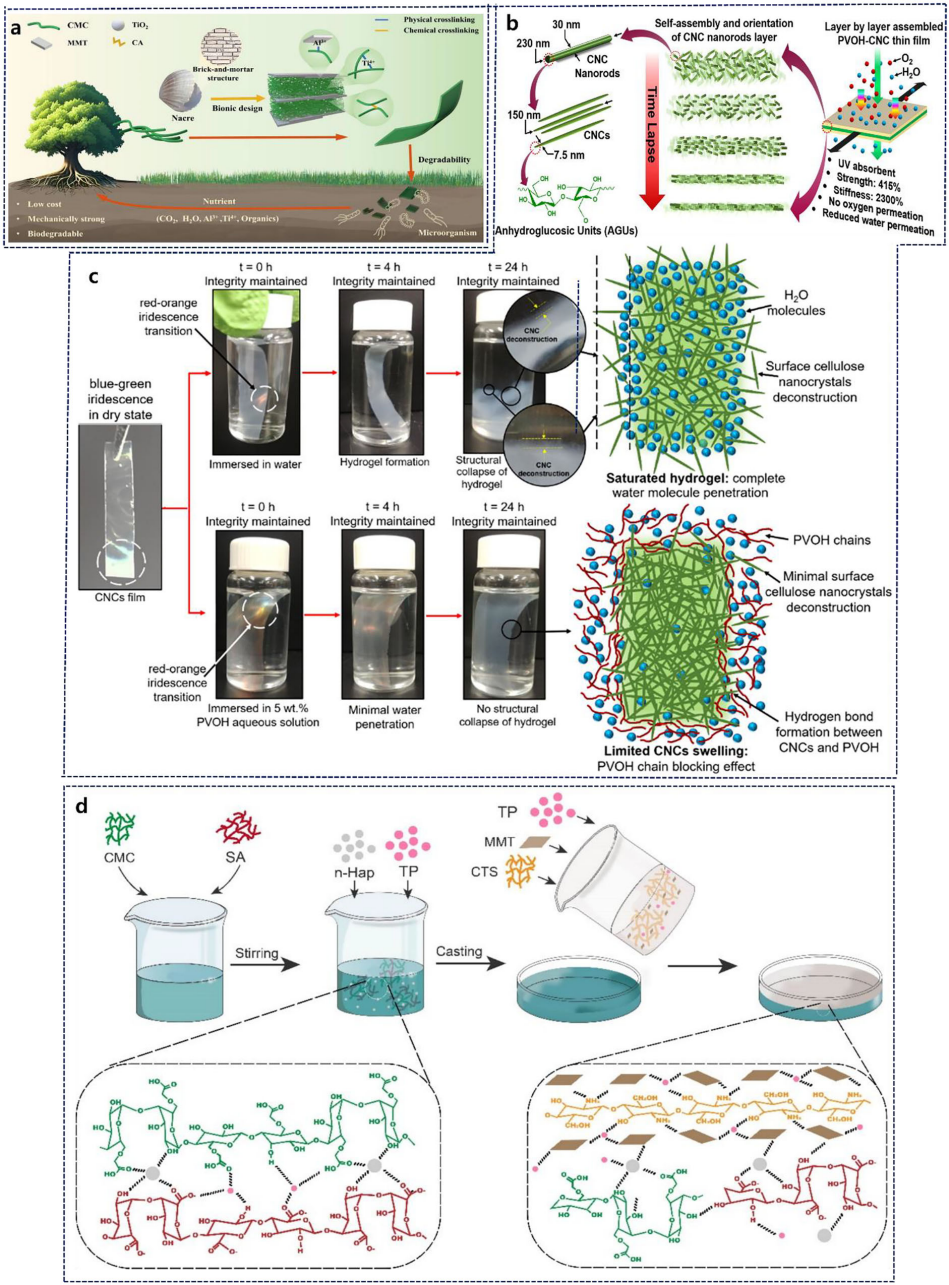
(a) Illustration of the bioinspired design strategy and the biodegradability of the carboxymethyl cellulose (CMC), nano‐montmorillonite (MMT), citric acid (CA), and TiO_2_‐based bioplastic (Liang et al. 2025). (b and c) Visualization of structural stabilization in self‐assembled cellulose nanocrystals (CNCs) and polyvinyl alcohol (PVOH)‐based multilayer films during 24‐h immersion in water, highlighting morphological transformations and the underlying mechanisms (Ogunsona and Mekonnen 2020). (d) Schematic of the fabrication steps and the architecture of the CMC/sodium alginate (SA)/nano‐hydroxyapatite (n‐HAP)/tea polyphenols (TP) and chitosan (CTS)/MMT/TP based dual‐layered composite film (Z. Wang 2025).

In another report, Liang et al. (2025) developed a multifunctional, water‐resistant, dual‐crosslinked nanocomposite film through the self‐assembly of CMC and nanomontmorillonite, establishing a chemically and physically double‐crosslinked network facilitated by TiO_2_ nanoparticles and citric acid (Liang et al. 2025) (Figure 7a). This structural mimicry improves interfacial interactions between organic and inorganic phases, resulting in enhanced mechanical and moisture‐barrier performance. The resultant bioplastic exhibited superior mechanical properties, achieving a tensile strength of 106.83 MPa, representing a 220.09% enhancement over a pure CMC‐based bioplastic and exceeding the tensile strength of alternative CMC‐based films. In addition to mechanical strength, it demonstrated remarkable water resistance (WA decreased to 42.88%), thermal stability, and UV protection. Solution casting facilitates the production of multifunctional packaging films with improved hydrophobicity, mechanical strength, and functional efficacy by optimizing surface roughness, interfacial chemistry, and filler dispersion. Although the technology is relatively simple, its ability to integrate structurally and chemically tailored nanocomponents renders it a fundamental technique for advancing next‐generation biodegradable food packaging materials (Z. Wang 2025).

### 6.2 LbL Assembly

Inspired by nature, LbL assembly entails the deposition of alternating layers of oppositely charged or complementary elements to construct multilayer nanocomposite films (Brown and Bhushan 2015). This method provides molecular‐level control of film composition and structure. For instance, Ogonsona et al. used hydrogen bonds between CNCs and PVA to fabricate composite films through LBL assembly. They found that at a CNC mass fraction of 10%, the strength and modulus increased by approximately 415% and 2300%, respectively, whereas oxygen permeation was completely blocked (Ogunsona and Mekonnen 2020). Immersion experiments confirm that PVOH restricts water infiltration into CNC films via hydrogen bonding, maintaining structural integrity and inhibiting interlayer mixing, thereby facilitating stable, well‐defined PVOH/CNC multilayers during LBL construction (Figure 7b,c). Z. Wang (2025) developed a dual‐layered CMC‐based film with enhanced water resistance and barrier properties using an LBL casting approach (Figure 7d). Featuring a nacre‐like upper layer and a dense bottom layer, the film achieved low moisture content (13.64%) and WS (6.26%), contributing to its superior water resistance. In addition, Yi et al. (2023) demonstrated that the performance of paper packaging was markedly improved by employing an LBL bio‐based coating consisting of chitosan, hydrophobically modified nanocellulose, and zein, which conferred oil resistance and water repellency and enabled adhesive‐free heat sealing (Yi et al. 2023). The resultant triple‐layer paper demonstrated a high kit number (12), a low Cobb60 value (2.6 g/m^2^), and substantial peel strength (372.6 N/m), along with enhanced mechanical qualities and decreased moisture and oxygen permeability. These stratified structures generate convoluted pathways for molecules, significantly reducing permeability. In terms of moisture, LbL films can be designed to have hydrophobic layers or cohesive hydrogen‐bonded networks that impede water vapor transport. The disadvantage of LbL is that it is a multi‐step, time‐intensive process that is inappropriate for large‐scale manufacturing (Xu et al. 2024). Nevertheless, it is a potent laboratory method to replicate intricate biological laminates and attain remarkable barrier efficacy in bio‐nanocomposites.

### 6.3 Electrospinning

**FIGURE 8:**
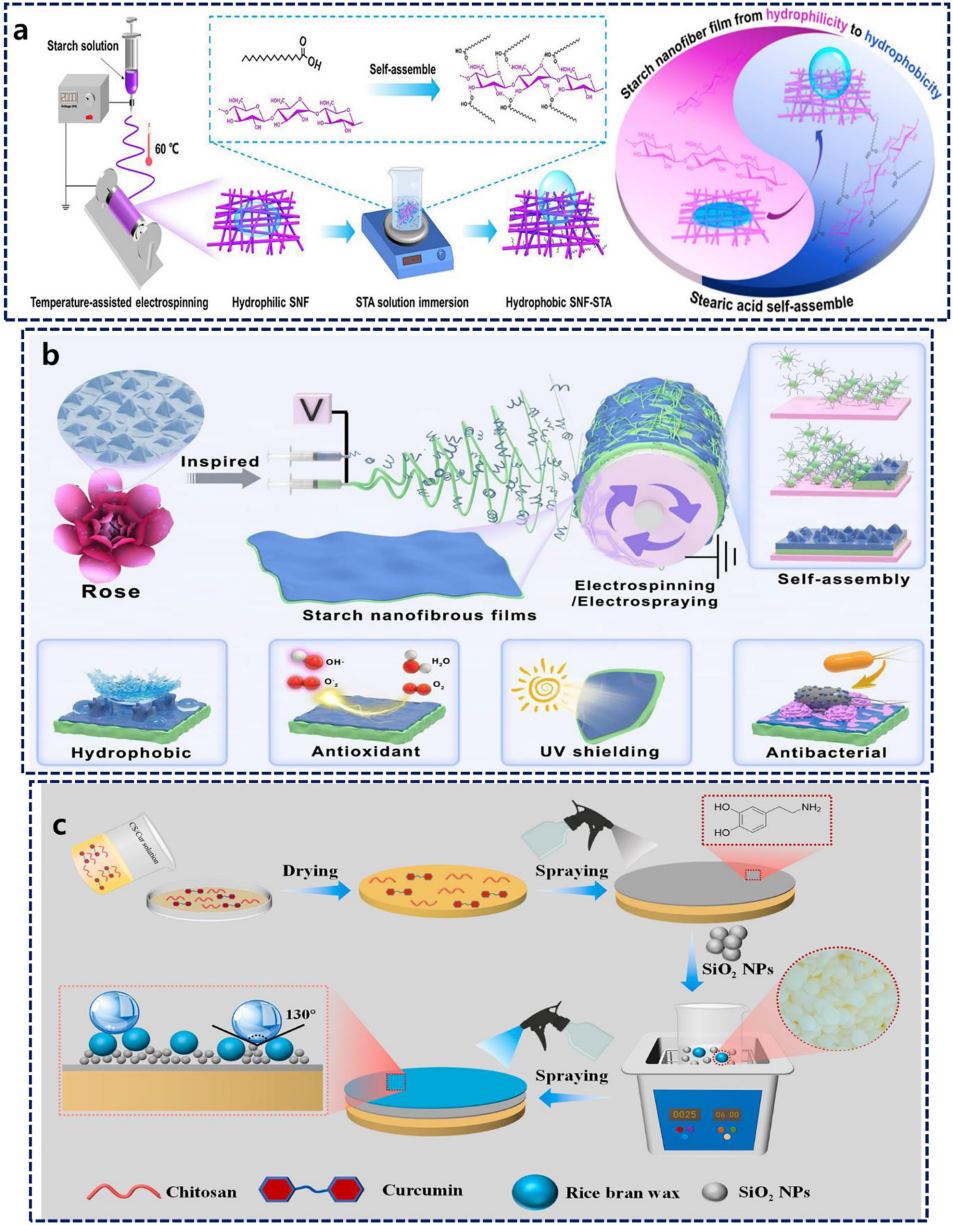
(a) Schematic representation of the fabrication process for a starch nanofibrous (SNF) and stearic acid (STA) film with a bioinspired hydrophobic surface (Cai et al. 2021). (b) Illustration of tannic acid‐driven interfacial self‐assembly forming multifunctional bioinspired SNFs (Xie, Feng, et al. 2024). (c) Fabrication pathway of a chitosan (CS) film coated with rice bran wax (RW) and SiO_2_ to achieve hydrophobicity (T. Liu et al. 2025).

Electrospinning generates nonwoven mats of ultrafine fibers by expelling a polymer solution through a needle under a strong electric field (L. Zhao et al. 2020). The resultant fibers, ranging from a few nanometers to several microns in diameter, aggregate to form a porous membrane with a large surface area. Electrospun biopolymer fibers (e.g., from starch, zein, and chitosan) have been investigated for active packaging and as hydrophobic coatings in food packaging. The intrinsic micro‐ to nanoscale roughness of fiber mats can confer water repellency, emulating the lotus‐leaf effect, provided the fibers have low surface energy or are subsequently coated with a hydrophobic coating. For instance, Cai et al. (2021) created a pure starch nanofibrous film (SNF) via temperature‐assisted electrospinning, devoid of nonstarch additives, and achieved hydrophobicity through the self‐assembly of STA using a solution‐immersion technique at 60°C for 1 h (Figure 8a). STA developed hierarchical flower‐like micro–nanostructures that reduced surface energy and improved thermal stability. Hydrophobicity was tunable with STA concentration, facilitating water roll‐off and self‐cleaning properties. In another report, Xie, Feng, et al. (2024) created SNFs covered with acylated tannic acid (ATA) by interfacial self‐assembly, drawing inspiration from hydrophobic surfaces resembling rose petals with adjustable water adhesion (Figure 8b). The SNFs/ATA attained a WCA of around 134.1°, a tensile strength of about 0.86 MPa, and a Young's modulus of roughly 43.48 MPa, while demonstrating antibacterial, antioxidant, UV‐blocking, and biodegradable characteristics. Xia et al. created a zein electrospun membrane incorporating peppermint oil (PO) and coated with methyltriethoxysilane (MTES). Hydrogen‐bonding interactions involving PO and the formation of Si–O–C bonds by MTES imparted pronounced hydrophobicity (WCA = 138° ± 5°) while preserving the membrane's bioactivity (Xia et al. 2023). These studies demonstrate that electrospinning, combined with bioinspired surface modifications, offers a versatile platform for producing hydrophobic, multifunctional, and biodegradable nanofibrous films. This strategy therefore holds considerable potential for next‐generation water‐resistant food packaging.

### 6.4 Freeze‐thaw (FT) Cross‐Linking

FT cross‐linking is a specialized method used to improve the water resistance and mechanical durability of hydrophilic biopolymer gels and films. This physical technique involves cyclic freezing and thawing of aqueous biopolymer mixtures such as PVA, gelatin, starch, or chitosan‐based composites, resulting in the formation of microcrystalline domains that serve as noncovalent physical cross‐linking sites (Bercea 2022). These domains strengthen the polymer matrix through hydrogen bonding and crystalline reorganization, thereby decreasing WS, enhancing dimensional stability, and improving barrier efficacy in humid conditions (D. Zhao et al. 2024). FT processing has emerged as an environmentally sustainable alternative to chemical cross‐linking, particularly suitable for biodegradable food packaging applications. Unlike covalent crosslinkers that may use hazardous chemicals or residual solvents, the FT technique relies solely on temperature‐induced phase changes, thereby conforming to clean‐label and sustainable packaging objectives (Ajdary et al. 2021). Wang, Fang, et al. (2025) reported that FT‐treated gelatin‐starch films exhibited enhanced hydrogen bonding and reduced moisture sensitivity (Wang, Fang, et al. 2025). Moreover, Wu, Lan, et al. (2025) indicated that FT‐aerogel films produced with ultrasonic assistance showed improved water barrier properties, making them suitable for high‐humidity conditions (Wu, Lan, et al. 2025). The cycle count generally ranges from 3 to 5, with more cycles strengthening the matrix, but excessive use may induce brittleness (Jia et al. 2023). FT cross‐linking is compatible with other processes, such as solution casting, facilitating incorporation into established packaging procedures (Yu, Hu, et al. 2025). Overall, FT cross‐linking offers a sustainable and scalable approach to customizing the characteristics of biodegradable packaging films, optimizing water resistance, mechanical strength, and environmental compatibility, essential attributes for food packaging in humid or refrigerated storage environments.

### 6.5 Sol–Gel Coatings

In the development of bioinspired food packaging materials, sol–gel coatings offer a versatile approach to enhance surface performance, particularly hydrophobicity and barrier efficacy. Sol–gel processing synthesizes inorganic or organic–inorganic hybrid networks from liquid precursors at low temperatures and has been used to enhance packaging films (Vartiainen et al. 2014). Alkoxysilane monomers generally undergo hydrolysis and condensation, forming a thin silica‐based layer on a biopolymer film. Functionalizing the sol–gel with hydrophobic groups, such as long‐chain alkylsilanes, yields a ceramic‐like covering that markedly reduces surface wettability (Vartiainen et al. 2014). Inspired by the naturally water‐repellent characteristics of lotus leaves and insect wings, sol–gel methodologies enable the creation of coatings that replicate these superhydrophobic structures on biodegradable substrates (Wang, Liu, et al. 2025). This entails using metal alkoxides, such as tetraethyl orthosilicate (TEOS) or MTES, under mild conditions to produce continuous silica‐based films (Hashjin et al. 2022). When modified with long‐chain alkyl or fluoroalkyl groups, these coatings yield low‐surface‐energy layers that mimic the hierarchical roughness and water‐repellency found in nature (Wang, Liu, et al. 2025). The sol–gel method has been extended to use biomass‐derived compounds in addition to mineral substrates. Yao et al. (2023) combined sol–gel chemistry with nanocellulose‐based films by submerging CNF/PVA‐coated wood panels in a TEOS/hexadecyltrimethoxysilane (HDTMS) solution (Yao et al. 2023). This treatment produced a coarse, silica‐based surface with low surface energy, resulting in a high WCA (166.8°) and self‐cleaning efficacy. The efficacy of this hybrid method underscores the compatibility of sol–gel techniques with bio‐derived substrates in the creation of entirely sustainable functional coatings. Moreover, Wang et al. (2022) enhanced the capabilities of biopolymer‐based materials by synthesizing a superhydrophobic starch‐based material using a sol–gel coating employing HDTMS and tetraethyl orthosilicate (TEOS) (F. Wang et al. 2022). The technique produced a low‐energy silica layer, with lyophilized starch contributing structural support and porosity. The resultant hydrophobic groups and micro–nano roughness resulted in a material exhibiting a high WCA (>153°), low surface area (<8°), and robust self‐cleaning and oil‐water separation capabilities. According to the preceding discussion, it is proposed that sol–gel technology may serve as a bioinspired, environmentally sustainable approach to improve biodegradable packaging. Emulating natural water‐repellent surfaces, it facilitates the creation of food‐safe films that exhibit enhanced hydrophobicity, durability, and barrier efficacy suitable for next‐generation applications.

### 6.6 Surface Modification and Biomimetic Coatings

In addition to bulk processing, optimizing the surface chemistry of biopolymer films is essential for achieving high water repellency. Chemical grafting techniques attach hydrophobic groups to polymer chains, thereby lowering the film's surface energy and water affinity. Esterifying starch, for example, by reacting hydroxyl groups with acyl chlorides or anhydrides, adds lipophilic side groups, resulting in starch esters that exhibit increased hydrophobicity and enhanced mechanical strength (Yang et al. 2024). Substituting long‐chain fatty acids (e.g., stearic or palmitic groups) on starch or cellulose significantly reduces WS and swelling, giving the biopolymer characteristics comparable to a hydrophobic thermoplastic (Yang et al. 2024). Modifications can be performed either in solution before film production or as a post‐treatment (surface grafting). Octenyl succinic anhydride (OSA)‐modified starch is amphiphilic and has been used in edible films to enhance moisture resistance while maintaining biodegradability (Punia et al. 2019). Cold plasma treatment is an environmentally sustainable, nonaqueous technique that alters the surface characteristics of biopolymer films while preserving their intrinsic qualities. Hydrophobic functional groups are grafted onto the film surface by exposing it to plasma gases (Hoque et al. 2022). This reduces surface energy and increases the WCA. Profili et al. (2023) used nontoxic organosilicon coatings on Kraft paper via atmospheric‐pressure dielectric barrier discharge plasma. The resultant hydrophobic coatings exhibited chemical stability when subjected to several food simulants (e.g., water, acetic acid, ethanol, and heptane), with minimal silicon migration, remaining below concentrations often observed in tap water. These results validate the chemical resilience and food safety of plasma‐deposited organosilicon coatings, endorsing their application in environmentally sustainable, hydrophobic packaging for wet or damp food items. Another approach is biomimetic hydrophobic coatings that replicate natural superhydrophobic surfaces. A notable example is the mussel‐inspired polydopamine coating: a thin poly(catecholamine) layer that attaches robustly to nearly any surface, mimicking mfps. Polydopamine coatings can serve as a reactive primer to adhere other molecules. T. Liu et al. (2025) employed this methodology to create a bioinspired hydrophobic coating (Figure 8c) by depositing a rice bran wax (RW)/SiO_2_ layer onto a chitosan–curcumin film matrix using dopamine‐assisted self‐polymerization, drawing inspiration from mussel adhesion. This coating generated hierarchical micro/nanostructures that markedly enhanced surface hydrophobicity, increasing the WCA from 84.28° to 130.43°. The coating exhibited superior performance (>118°) despite mechanical damage and chemical exposure. Furthermore, it enhanced mechanical durability and decreased WVP. The catechol‐based adhesive ensured the waxy layer's attachment, resulting in a bilayer film with a hydrophilic bottom (for breathability and mechanical strength) and a hydrophobic upper surface (for water resistance). Analogously, coatings of natural waxes or fatty acid complexes, modeled after insect cuticles, have been used on polysaccharide films to protect them from moisture. Bioinspired surface treatments can significantly elevate WCAs to the 120°–160° range (superhydrophobic), exemplified by chitosan films coated with nanosilica and PDMS, which inhibited liquid food (juices, yogurt) from wetting (WCA 153°) or adhering to the surface (Yu, Sun, et al. 2025), underscoring its potential for anti‐adhesion applications in liquid food packaging. Overall, surface modification techniques, such as chemical grafting, plasma treatment, and biomimetic coatings, significantly enhance the water resistance of biopolymer films, enabling durable, hydrophobic surfaces suitable for eco‐friendly food packaging applications.

## 7 Applications in Food Packaging

### 7.1 Moisture Control and Shelf Life Extension of Foods

Mitigating undesirable moisture transfer is essential for preserving many food items, including meat, seafood, fresh fruit, vegetables, dairy, and baked goods. Excess moisture frequently hastens microbiological deterioration and compromises texture. For instance, humidity causes crunchy snacks to become mushy and reduces their shelf life (Hu et al. 2025). By modulating surface wettability and establishing hydrophobic barriers, bioinspired packaging can preserve the product's necessary microenvironment. This approach impedes microbiological proliferation, maintains food desiccation, and markedly prolongs shelf life while concurrently reducing food waste due to liquid deterioration or adhesion (Hu et al. 2025). Table 2 shows recent developments demonstrating bioinspired contributions to the advancement of water‐resistant food packaging solutions. Recent studies clearly demonstrate these benefits across various food categories, as discussed in Table 2.

**TABLE 2:** Bioinspired water‐resistant sustainable packaging solutions for various food products.

Bioinspired object	Materials and packaging format	Hydrophobic properties	WVP and water‐resistance performance	Mechanical performance	Application	Scalability	Refs.
**Lotus leaf**	Honeycomb wax superhydrophobic top layer + edible interlayer of arabic gum/gelatin/glycerine; spray‐coated onto plastic, polypropylene, or glass substrates	WCA ∼ 157.2°; strongly repels milk, yogurt, and honey	—	Compressive stress >0.18 MPa and TS ∼30 kPa	Functional inner packaging interface to reduce residual liquid foods like milk, yogurt, and honey	High: simple, low‐cost microemulsion spray coating process described as scalable and suitable for large‐area fabrication; applicable to flexible plastic substrates	Y. Li et al. (2018)
**Lotus leaf**	Soybean polysaccharide (SPS)/SPS‐stabilized AgNPs/gelatin/beeswax/Laponite clay/glycerol composite film with a carnauba wax‐coated lotus‐like superhydrophobic front surface; asymmetric Janus film	The water contact angle of the Janus film reached 157.2°	A WVP of 3.6–4.1 × 10^−10^ g/m/s/Pa was reported for the film series. L‐SPGBC water solubility (WS) was only 16.8% ± 1.1% vs. 98.0% ± 0.9% for neat SPS	TS = 8.91 Mpa (4.61 MPa for neat SPS), elastic modulus = 250.97 MPa; reduced EAB compared with gelatin‐containing film with retained flexibility	Successfully preserved chicken, pork, and grapes, reducing bacterial growth, water loss, discoloration, and decay during 7 days of storage	Moderate: casting and wax coating are scalable, but surface replication and nanoparticle dispersion require process control	Z. Ma et al. (2023)
**Lotus leaf**	PVA/chitosan (PVA/CS) composite film substrate coated with beeswax and SiO_2_ by spray drying. Beeswax only and beeswax/SiO_2_ formulations were also studied	Beeswax‐coated PVA/CS film: ∼152.1°, Beeswax/SiO_2_‐coated PVA/CS film: ∼156.2°; WCA remained >150° after 10 min	WVTR of 689.83, 306.88, and 350.40 g/m^2^ 24 h^−1^ for PVA/CS, Beeswax‐coated PVA/CS, and Beeswax/SiO_2_‐coated PVA/CS films, respectively	TS values of 44.7, 37.7, and 38.3 MPa, with corresponding EAB of 157.0%, 158.9%, and 157.8% for neat PVA/CS, beeswax‐coated PVA/CS, and beeswax/SiO_2_‐coated PVA/CS films	Proposed for the Antibacterial‐adhesion/self‐cleaning food packaging surfaces	Promising: simple spray‐drying/spray‐coating process using inexpensive materials. Successfully applied not only to PVA/CS but also to conventional PE and PET films, which became superhydrophobic	H. Huang et al. (2022)
**Lotus leaf**	Paper substrate dip‐coated with a CNC‐stabilized pickering emulsion containing chitosan (CS) in the aqueous phase and acrylic rosin (AAR) in the oil phase, followed by spraying hydrophobic SiO_2_ nanoparticles (hSiO_2_)/PDMS	A WCA of 155.8° with self‐cleaning and antifouling behavior	WVP = 1.42 × 10^−10^ g/m/s/Pa, an 80.7% reduction from uncoated paper. Paper boats floated for 60 days	Dry TS increased from 10.01 MPa (uncoated) to 21.67 MPa. Wet TS after 6 h water exposure: 20.35 MPa vs. 5.50 MPa for uncoated paper	Water‐ and grease‐resistant food packaging: paper cups for water, sprite, tea, milk and coffee. No leakage after 6 h and lower liquid residue than commercial cups	Promising: Dip‐coating + spray coating uses relatively low‐cost materials and was successfully applied to filter, copy, kraft and butcher papers	Lu et al. (2025)
**Lotus leaf**	Bilayer active film. Outer layer: chitosan (CS) + ZnO/TiO_2_ nanoparticles, molded using a lotus leaf‐derived PDMS template. Inner layer: sodium alginate (SA) + citral‐loaded zein nanoparticles coated with a fisetin‐Fe^3+^ metal‐phenolic network (Z‐CA@MPN)	Lotus‐like outer surface showed WCA >130°; Final film WS 28.3% ± 1.5% and moisture absorption remained <40% during the test	WVTR = 289.3 ± 1.5 g/m^2^ 24 h^−1^, vs. 327.0 ± 2.6 g/m^2^ 24 h^−1^ for CSM/SA	TS = 29.3 ± 3.6 MPa (∼38% above CSBM/SA); toughness = 191.6 ± 23.5 J/m^3^; EAB >2× that of CSBM/SA	Active packaging for fresh‐cut apples and lotus roots. Reduced browning, weight loss, hardness deterioration, and microbial growth during 4 days at 4°C	Currently limited to laboratory scale	Wei et al. (2025)
**Lotus leaf**	Janus bilayer electrospun fibers. Hydrophilic KA layer: konjac glucomannan (KGM) + tea polyphenols (TP) + anthocyanin/curcumin/tannic‐acid–Fe^3+^ polyphenol particles (ACT NPs). Hydrophobic PSA layer: PLA + stearic acid (SA)‐modified ACT nanoparticles (SA‐ACT NPs)	PSA‐facing surface: WCA = 154.5°, remaining unchanged for 120 min, indicating stable superhydrophobicity	WVP ≈ 2.6 g mm/m^2^/day/kPa for KA/PSA; and lowest among tested fibers. Water sealed with KA/PSA showed essentially no mass loss over 120 h	TS of ≈7.2 MPa and EAB of ≈18% was observed for KA/PSA	Multifunctional waterproof active packaging for fresh‐cut pakchoi and potatoes. Reduced yellowing/browning, weight loss and microbial growth and better maintained firmness/TSS during 10‐day storage	Low to moderate: Fabrication used laboratory sequential electrospinning plus multistep ACT‐NP synthesis, 3‐day dialysis and freeze‐drying	Wu, Wang, et al. (2025)
**Lotus leaf**	Superhydrophobic surface coating composed of beeswax (BW) + bacterial cellulose nanofibrils functionalized with food‐grade SiO_2_ (BCn–SiO_2_)	WCA = 153°; repelled honey, yogurt, and chocolate sauce	—	Excellent mechanical durability: hierarchical structure retained after 10 sandpaper‐abrasion cycles, scratching, and repeated tape peeling	Anti‐residue/self‐cleaning food contact packaging coating. Repelled coffee, yogurt, colored water, chocolate milk, honey and chocolate coating	Limited to moderate potential; industrial scale not demonstrated	Frota et al. (2024)
**Canna leaf**	Three‐layer hierarchical intelligent colorimetric sensing film/smart label based on regenerated cellulose (RC), in‐situ ZnO nanoparticles, Arnebia euchroma naphthoquinone dyes (AENDs), and SA	WCA = 155.1°; Hierarchical SA nanoflowers/nanoflakes (∼496.7 nm) create the superhydrophobic surface. Retained WCA 153.5° after 240 days at room temperature and 151.0° after 24 h at 60°C	WS of 7.12% ± 0.51% (RC) vs. 0.92 ± 0.31%; swelling index <115%, with dimensional stability under high humidity	TS of ∼105.07 MPa and shear modulus of ∼17.28 GPa	Intelligent freshness monitoring label for shrimp and pork	High/potentially scalable: Roll‐to‐roll fabrication and solvent‐evaporation processing were presented as scalable manufacturing routes	H. Dong et al. (2023)
**Rose petal**	Starch nanofibrous film (SNFs) fabricated by electrospinning and coated with acylated tannic acid (ATA) through electrospraying/interfacial self‐assembly	WCA ≈ 134.1° for SNFs/ATA‐10 vs. ∼0° for uncoated SNFs. Food liquid CA was 100.5° for milk, 109.7° for orange juice, 98.7° for coffee, and 128.2° for electrolyte drink	WVP of 362.2 ± 7.49 g mm/day/m^2^/kPa, compared with 422.6 ± 8.08 for the control	TS ≈ 0.86 MPa, nearly 2× neat SNFs; Young's modulus ≈43.48 MPa, ∼561.8% increase	Active fruit packaging: Extended cherry tomato shelf life to 15 days	Good/high scale‐up potential: electrospinning and ATA electrospraying/electrospraying suitable for large‐scale production	Xie, Feng, et al. (2024)
**Mussel‐inspired**	Superhydrophobic active paper prepared by coating paper first with mussel‐inspired polydopamine (PDA), followed by halloysite nanotubes (HNTs) + PDMS + SA	A WCA of 157.5° and food liquid WCAs of 151.1°–157.6° for tea, coffee, orange juice, cola, milk, yogurt, honey and ketchup	WVP decreased by 19.5% vs. original paper (∼5.1 × 10^−10^ g/m/s/Pa)	TS increased by 89.9% and EAB by 22.3% vs. original paper	Multifunctional active fruit/food packaging: delayed cherry tomato softening during 7 days of ambient storage	Moderate: industrial scale not demonstrated	F. Yu et al. (2023)
**Mussel‐inspired**	Hydrophobic intelligent indicator film: chitosan (CS) + curcumin (Cur) matrix, dopamine/PDA adhesive interlayer, and sprayed rice bran wax (RW) + hydrophobic SiO_2_ coating	The WCA increased from 84.28° to 130.43°; and food liquids showed CA >120°–125°	WVP was the lowest among samples and decreased by 0.19‐fold vs. CC	TS decreased from 61.45 MPa (CC) to ∼50 MPa (CC‐R‐S), while EAB increased 1.73‐fold	Intelligent/active pork packaging: Monitored pork freshness and prolonged acceptable pork storage by approximately 2 days at both 25°C	Moderate: Spray coating and dopamine deposition are adaptable to roll‐to‐roll processing, but nanoparticle dispersion and coating uniformity require control	Liu et al. (2025)
**Mussel‐inspired**	Low methoxyl pectin (LMP) covalently grafted with dopamine hydrochloride (DA) through EDC/NHS‐mediated amide coupling	—	WVP decreased from 2.0 × 10^−12^ to approximately 1.2 × 10^−12^ g/cm/s/Pa, depending on formulation	TS increased from 10.90 MPa for pectin to ∼15.43–24.00 MPa after dopamine modification	Active edible coating for banana preservation: Delayed visible browning/spot formation and extended shelf life to approximately 9 days	Low to moderate scalability; EDC/NHS coupling, inert conditions, 72‐h dialysis, and freeze‐drying process limit high volume production	L. Li et al. (2026)
**Mussel‐inspired**	Inner layer: chitosan grafted with hydrocaffeic acid (HCA) by free radical grafting (R‐CS). Outer layer: sodium alginate (ALG) + 3 wt% LDHs@MPN, where LDHs are coated with a quercetin‐Cu^2+^ metal‐phenolic network	Alginate layer WCA increased from 47.56° to 117.67°; final bilayers showed WCAs of ∼ 69.37–76.2°, indicating moderate wettability rather than superhydrophobicity	Water absorption decreased after bilayer formation (RLA‐3 showed the lowest value of ∼18%, vs. ∼27% for ALG)	TS reached 14.40 vs. 4.48 MPa for ALG (∼ 221% improvement), and elongation at break was ∼27.80% vs. 42.80% for ALG	Active strawberry preservation coating: Prevented visible mold and severe shriveling of strawberries for 7 days	Low to moderate: multistep grafting, MPN functionalization, dialysis, and freeze‐drying increase process complexity	Quan et al. (2026)
**Mussel‐inspired**	Hydrophobic ethyl cellulose (EC) outer layer + electrospun gelatin/chitosan/glycerol/TA inner layer	—	Electrospun films exhibited better moisture resistance than cast films	ES‐double showed the highest TS (6.97 MPa vs. 3.14 MPa for ES‐single‐TA), while LbL‐double exhibited the highest EAB (65.34%)	Proposed as multifunctional food packaging film; especially suitable for light‐sensitive foods and potential intelligent‐packaging platforms	Moderate: LbL casting is described as simple and scalable, but electrospinning has processing complexity	Jin et al. (2026)
**Nacre‐inspired**	CMC matrix containing exfoliated g‐C_3_N_4_ nanosheets, citric acid crosslinks, and biomass‐derived carbon dots	—	WVP of 2.0 ± 0.2 × 10^−9^ (CMC) to 1.2 ± 0.2 × 10^−9^ g m/m^2^/Pa/s, film retained its shape after 100 h in water	TS increased from 40.8 ± 1.1 to 64.8 ± 1.9 MPa; modulus increased to approximately 2.2 GPa	Active high humidity fruit packaging: Maintained blueberry quality up to 16 days at refrigerated storage	Low‐moderate scalability: high temperature g‐C_3_N_4_ synthesis, acid exfoliation, and dual cross‐linking increase process complexity	Khan et al. (2026)
**Duck feather**	CMC/gelatin (1:1), resveratrol (Res); duck‐feather texture replicated using a PDMS template, followed by candelilla‐wax spray coating	An Optimal WCA ∼142.57°, compared with 80.34° for CMC/Gel; identical wax‐coated but untextured film: 121.57°, Food‐liquid CA >120°	WVP ≈ 4.1 × 10^−11^ vs. 7.33 × 10^−11^ g/Pa/s/m for CMC/Gel	TS ≈ 23 MPa; EAB ≈ 56% vs. 15.30 MPa and 42.48% for neat CMC/Gel	Active + intelligent packaging: Enabled freshness indication, antioxidant and antibacterial protection	Low‐moderate: PDMS feather templating, repeated cyclohexane‐based wax spraying, and higher cost than plastic increase process complexity	Wang, Jiang, et al. (2025)
**Swan feather**	CMC/PVA (1:1), quercetin (QR); feather‐like structure replicated using a PDMS template and coated with carnauba wax (CW) by spraying	WCA = 138°, vs. 26° for QR‐containing uncoated film and 114.5° for wax‐coated non‐bionic surface; natural swan feather: 153°	Reported WVP ≈ 1250.0 g/m^2^/day for C‐CPQ1, substantially below CPQ1 (2655.13 g/m^2^/day)	A TS of ≈9 MPa, EAB ≈44% vs. 30.75 MPa and 35.38% for neat CMC/PVA	Active/intelligent packaging: Monitored meat freshness and extended acceptable storage by 2 vs. polyethylene packaging	Low to moderate: Film casting is scalable, but feather‐templated structuring and wax deposition increase manufacturing steps	Jiang et al. (2024)
**Insect inspired**	Chitosan + gelatin + porous shellac hydrogel microparticles (SH; 0.1%–0.5%)	WCA increased from ∼13.2° (chitosan) to 78.7° (0.5% SH)	WVP increased with SH content (0.49 vs. ∼0.34 g mm/m^2^/h/kPa for 0.1% SH/C and chitosan), while WS decreased from ∼44% to ∼40% (0.1% SH) and ∼28% (0.5% SH).	TS remained ∼8.8 MPa, comparable to neat chitosan; EAB decreased from 47.6% to 31% and puncture strength from 1896 to 1296 N/mm at 0.5% SH	Passive MAP for fresh‐cut onions, leeks and spinach. Controlled CO_2_, O_2_, water vapor, and ethylene transfer; preserved fresh‐cut vegetables	Moderate scalability: simple casting/particle incorporation, but shellac‐hydrogel freeze‐drying increases time and cost	J. Ma et al. (2024)
Composite Lotus leaf + nacre‐inspired	Poly(vinyl alcohol) (PVA), cellulose nanofibrils (CNF), 3‐glycidyloxypropyltrimethoxysilane (KH560), and methyltrimethoxysilane (MTMS) (MK‐CNF@PVA coated onto K‐CNF@PVA)	WCA = 116.65° for final MK‐K‐CNF@PVA; hydrophobic MK‐CNF@PVA layer reached 118.96°. Contact angle decreased only 7.4% after 10 min	WVTR = 0.4761 g/m^2^/day vs. ∼1.1–1.2 g/m^2^/day for pure PVA at 50% RH	TS 110.98 MPa and EAB 216.09% vs. ∼42.5 Mpa and ∼230%–235% for pure PVA	Fruit packaging as well as applicable as a high‐barrier coating for paper packaging	High scalability potential: one‐step aqueous silane modification and kraft‐paper coating are favorable, though CNF processing and prolonged drying require optimization	Deng et al. (2026)

Abbreviations: PE, polyethylene; PET, polyethylene terephthalate; PLA, polylactic acid; PVA, polyvinyl alcohol; WCA, water contact angle; WVP, water vapor permeability; WVTR; water vapor transmission rate.

#### 7.1.1 Water‐Resistant Active and Intelligent Films for Meat and Seafood Packaging

High‐protein food products such as meat and fish deteriorate quickly when exposed to moisture and microorganisms. Bioinspired moisture barrier coatings have demonstrated significant efficacy in prolonging the shelf life of such products. A composite inspired by swan feathers (CMC + PVA reinforced with natural carnauba wax) mimics the feather's multilayered waterproof packaging film. The resultant film exhibits a WCA of around 138° (comparable to genuine feathers) and performs effectively in real storage tests: Packaged pork remains fresh for 5 days under refrigeration, whereas pork in regular packaging spoils in ∼2 days (Jiang et al. 2024). This approximately 3‐day enhancement is attributed to the film's ability to maintain a drier meat surface and inhibit bacterial proliferation, facilitated by the waxy hydrophobic coating.

Furthermore, the duck feather mimic film (CMC/gelatin with candelilla wax) achieves similar improvements for red meat. Beef steaks packaged with this composite film remain fresh for approximately 5 days, which is roughly 2 days longer than when stored in normal PE wrap (Wang, Jiang, et al. 2025). These packaging approaches not only block external moisture but also inhibit drip loss from the meat, preserving juiciness while preventing exudate accumulation that may promote bacterial growth. Additionally, a bioinspired nanocomposite film composed of pectin and gelatin, infused with 5 wt% TiO_2_ nanoparticles and curcumin, develops a water‐resistant, oxidation‐inhibiting packaging solution for salmon fillets. The active layer reduces moisture penetration and oxidative rancidity, prolonging the shelf life of salmon by about 6 days, with the 5 wt% TiO_2_ formulation achieving preservation of up to 12 days (Candra et al. 2023). It should be noted that TiO_2_ nanoparticles present a distinct regulatory consideration. In 2022, the EFSA concluded that TiO_2_ could no longer be considered safe as a food additive (E171) due to potential genotoxicity concerns, leading the European Union to withdraw its food additive approval (European Commission 2022; Younes et al. 2021). However, the use of TiO_2_ in food packaging differs from its direct addition to food, as it is incorporated within the polymer matrix and must comply with established migration limits to ensure minimal transfer into food (Alizadeh Sani et al. 2022; Younes et al. 2021). In the United States, TiO_2_ remains permitted for food contact applications under 21 CFR 178.3297, subject to compositional and migration constraints (U.S. Food and Drug Administration 2024). These differing regulatory treatments underscore the need to evaluate TiO_2_‐containing packaging films specifically for nanoparticle migration rather than relying on its historical food additive approval status.

**FIGURE 9:**
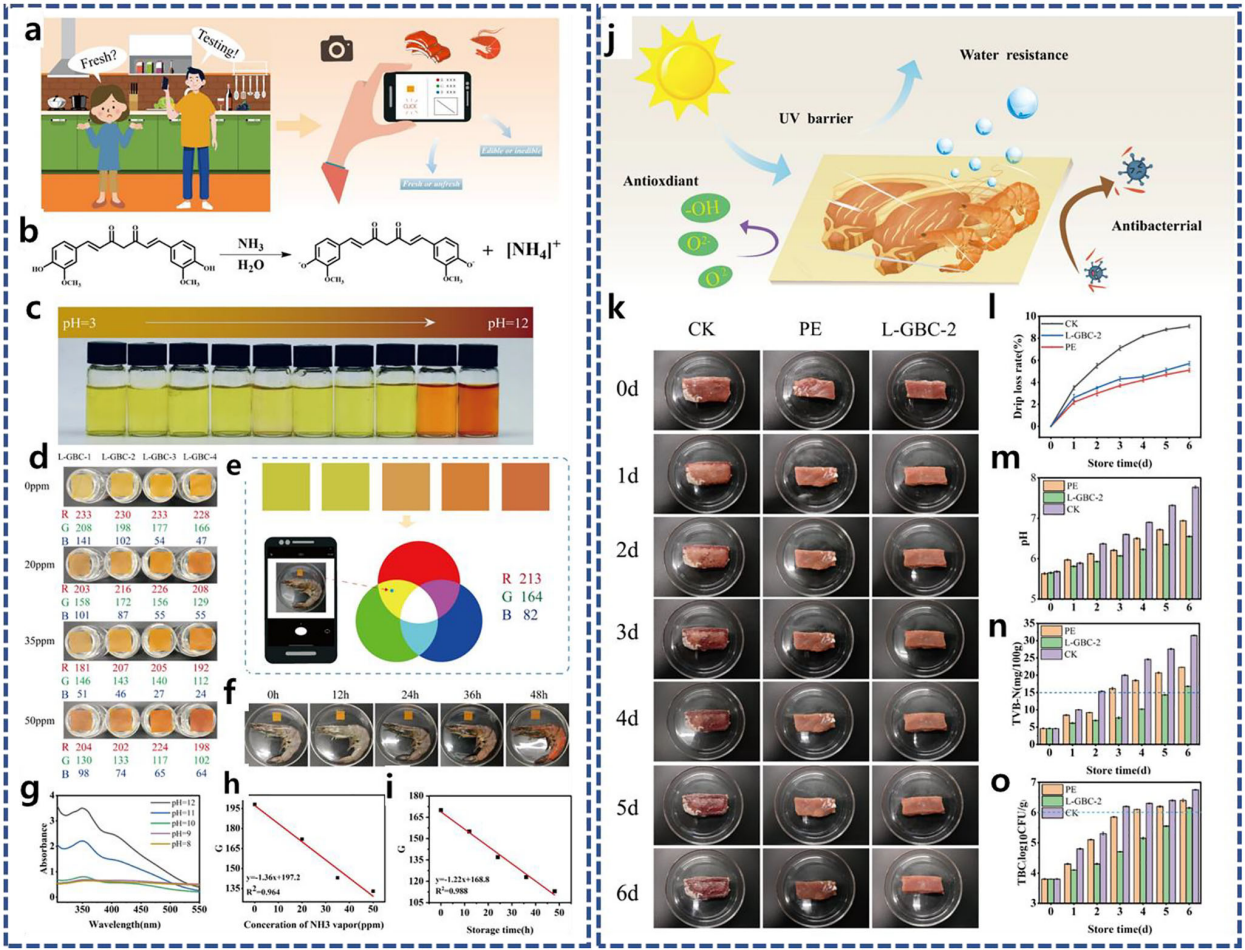
Visual monitoring and preservation performance of gelatin‐bacterial cellulose–curcumin composite (L‐GBC) films: (a) film application for freshness detection; (b and c) curcumin's structural response and visible color shift under alkaline/ammonia conditions; (d) *R*, *G*, *B* values trends under varying ammonia vapor levels; (e and f) schematic and images of shrimp spoilage detection; (g–i) UV–Vis spectrum and quantitative correlation between ammonia levels, *G* value, and storage time. (j) Proposed preservation mechanism; (k–o) evaluation of pork quality, including visual appearance, drip loss, pH, total volatile basic nitrogen (TVB‐N), and total bacterial count (TBC). Reprinted from Jiang et al. (2025).

In contrast, the above‐discussed findings validate that moisture‐resistant biopolymer films, particularly when integrated with natural antimicrobials or antioxidants, can significantly maintain the quality of meat and seafood. By maintaining low water activity on the product surface and inhibiting oxidative spoilage, the coatings impede bacterial growth and chemical breakdown that would otherwise compromise the safety of protein meals. Conversely, bioinspired design has facilitated the creation of water‐resistant films that are not only exceptionally water‐repellent but also include active preservation properties and intelligent features for sensing or reporting food quality. Contemporary packaging employs active films that intentionally incorporate functional ingredients, such as antimicrobials and antioxidants, to mitigate deterioration, whereas intelligent films incorporate indicators or sensors to assess freshness (Zhong et al. 2025). Integrating these principles with bioinspired water‐resistant materials, next‐generation films can safeguard food from moisture, prolong shelf life, and offer real‐time quality feedback. An exemplary case is the recently documented lotus‐leaf‐inspired gelatin/bacterial cellulose film infused with curcumin (L‐GBC), a natural antioxidant and pH‐sensitive substance. This all‐natural composite was designed with a lotus‐inspired micro–nanostructured surface, significantly enhancing its hydrophobicity and water vapor barrier, thereby addressing the intrinsic water sensitivity of its biopolymer constituents (Jiang et al. 2025). The L‐GBC films, containing 0.1% curcumin, demonstrate a pH‐responsive color shift for real‐time monitoring of food freshness in intelligent packaging applications (Figure 9a–i). They facilitate ammonia detection by smartphone RGB analysis, demonstrating significant linearity with deterioration levels. This intelligent packaging film provides an effective, economical method for monitoring freshness in perishable items such as shrimp. Moreover, as depicted in Figure 9j, the mechanism of active packaging demonstrates that the material systematically blocks external contaminants by incorporating hydrophobic properties alongside synergistic antibacterial, antioxidant, and anti‐UV effects, thereby significantly prolonging the shelf life of food. It exhibits water resistance, scavenges free radicals (antioxidant properties), inhibits microbial growth, and indicates deterioration through pH‐responsive color and fluorescence alterations. In practical evaluations, this L‐GBC bioinspired film preserved hog meat for around 5 days, which is roughly 2 days longer than traditional PE packaging (Figure 9k–o). These results illustrate how a single biomimetic material may function as a waterproof active package (preventing moisture infiltration and microbiological proliferation) and an intelligent freshness indicator to enhance food safety. Comparable multipurpose films have been created by emulating many natural models. A CMC/gelatin film, inspired by duck feathers, incorporated natural additives such as resveratrol (a polyphenolic pH‐sensitive active compound) and candelilla wax to achieve a WCA of approximately 142°, while also providing antioxidant and antimicrobial properties and responding to spoilage volatiles. This hydrophobic intelligent/active film enabled timely quality monitoring and extended the shelf life of beef by almost 2 days compared to conventional packaging (Wang, Jiang, et al. 2025). The incorporation of Res and candelilla wax exerted little influence on the deterioration of the films, whereas the composite film showed remarkable biodegradability.

**FIGURE 10:**
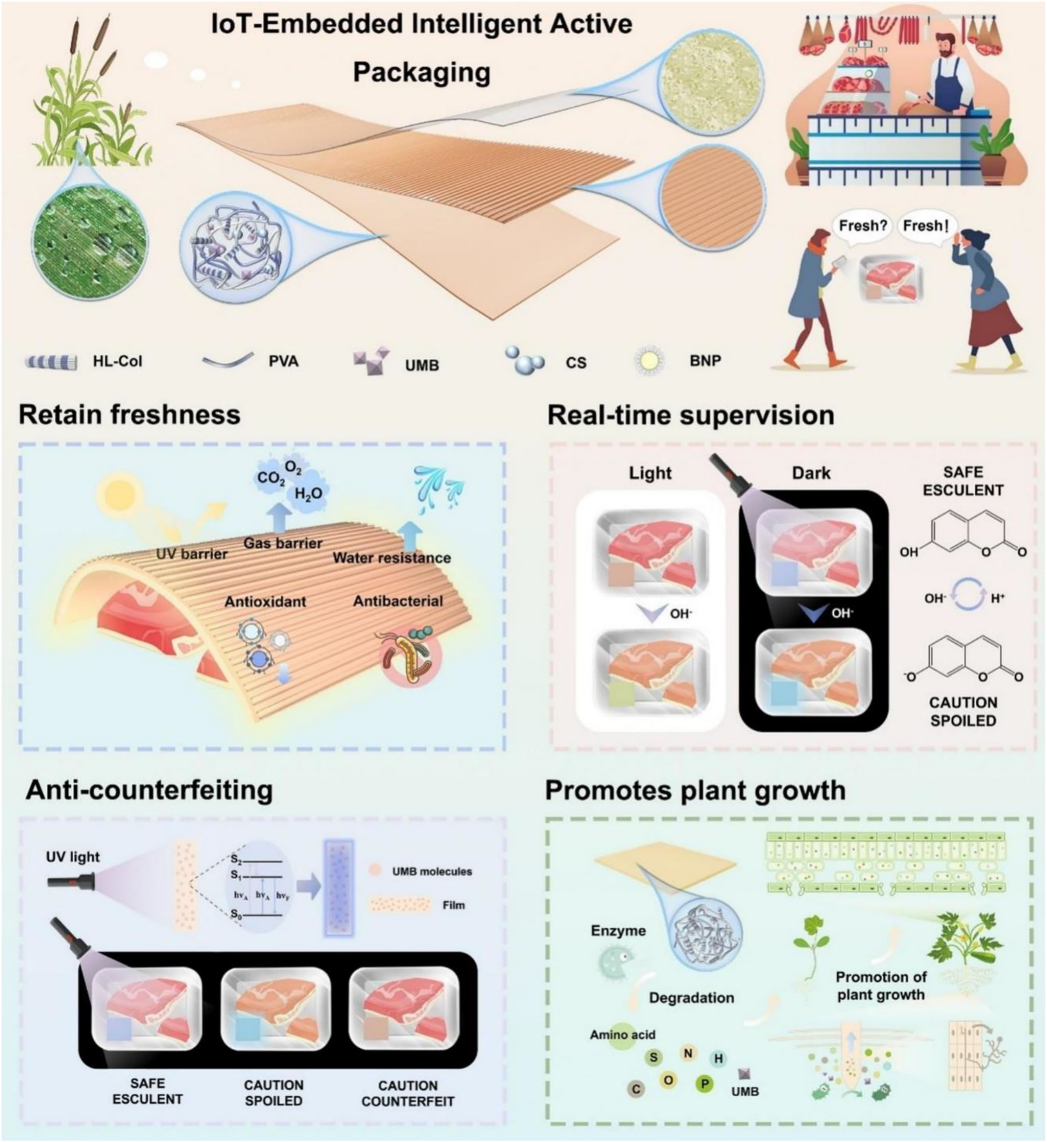
Schematic illustration of the architecture, functional features, and practical uses of the reed‐leaf‐inspired hierarchical‐structured composite platform (HPCU‐B) developed using HL‐Col, polyvinyl alcohol (PVA), chitosan (CS), and umbelliferone (UMB) (Li, Luo, et al. 2025).

Likewise, a mussel‐inspired chitosan nanocomposite coating, supplemented with hydrophobic SiO_2_ and curcumin, achieved a WCA above 130° and extended the shelf life of pork by 2 days at both ambient and refrigerated temperatures (T. Liu et al. 2025). The incorporation of curcumin improved water resistance by creating a denser, less permeable film network, while also providing antioxidant activity and a modest pH‐indicating function. Mussel‐inspired catechol chemistry further enhanced adhesive cohesion, contributing to the formation of a durable and bioactive coating (T. Liu et al. 2025). In another report, Li, Luo, et al. (2025) developed a bioinspired packaging film (HPCU‐B) modeled after reed leaves, which had a hierarchical surface structure that markedly improved hydrophobicity, elevating the WCA from 92.1° to 139.2°. The HPCU‐B matrix, consisting of HL‐Col, PVA, and chitosan (CS), achieved synergistic polymer entanglement via hydrogen bonding and electrostatic interactions, enhancing its mechanical strength and structural integrity. This superhydrophobic design offered robust moisture resistance, UV protection exceeding 98%, and gas barrier characteristics. In shrimp preservation experiments, HPCU‐B prolonged shelf life by 2 days and facilitated real‐time spoiling detection through pH‐responsive color and fluorescence changes. The entirely bio‐based, biodegradable polymer offers a multipurpose, sustainable solution for intelligent food packaging applications (Figure 10). Furthermore, Wang, Jia, et al. (2025) created a bioinspired multifunctional packaging film (CW‐CPM) made from carnauba wax, CMC, PVA, and myricetin. The film emulated the textures of peacock feathers and bamboo, attaining superhydrophobicity (contact angle 139.4°) and improving its moisture barrier efficacy. Besides hydrophobicity, the film demonstrated UV‐induced reversible fluorescence and a dynamic color response to biogenic amines, facilitating real‐time spoiling detection. The film accurately detected spoiling inflection points (≥3.25 days) in goat meat, graded freshness phases, and extended shelf life through its barrier and antioxidant properties, showcasing significant promise for intelligent, moisture‐resistant food packaging. These examples underscore that bioinspired water‐resistant packaging films can be synergistically integrated with active agents (e.g., essential oils, plant polyphenols, and antimicrobial nanoparticles) and smart indicators (pH‐sensitive dyes, fluorescence tags) to produce multifunctional packaging. Such films keep food surfaces dry and clean, inhibit spoilage organisms, and alert consumers to freshness, addressing multiple challenges in food packaging simultaneously.

#### 7.1.2 Fresh Fruits and Vegetables

**FIGURE 11:**
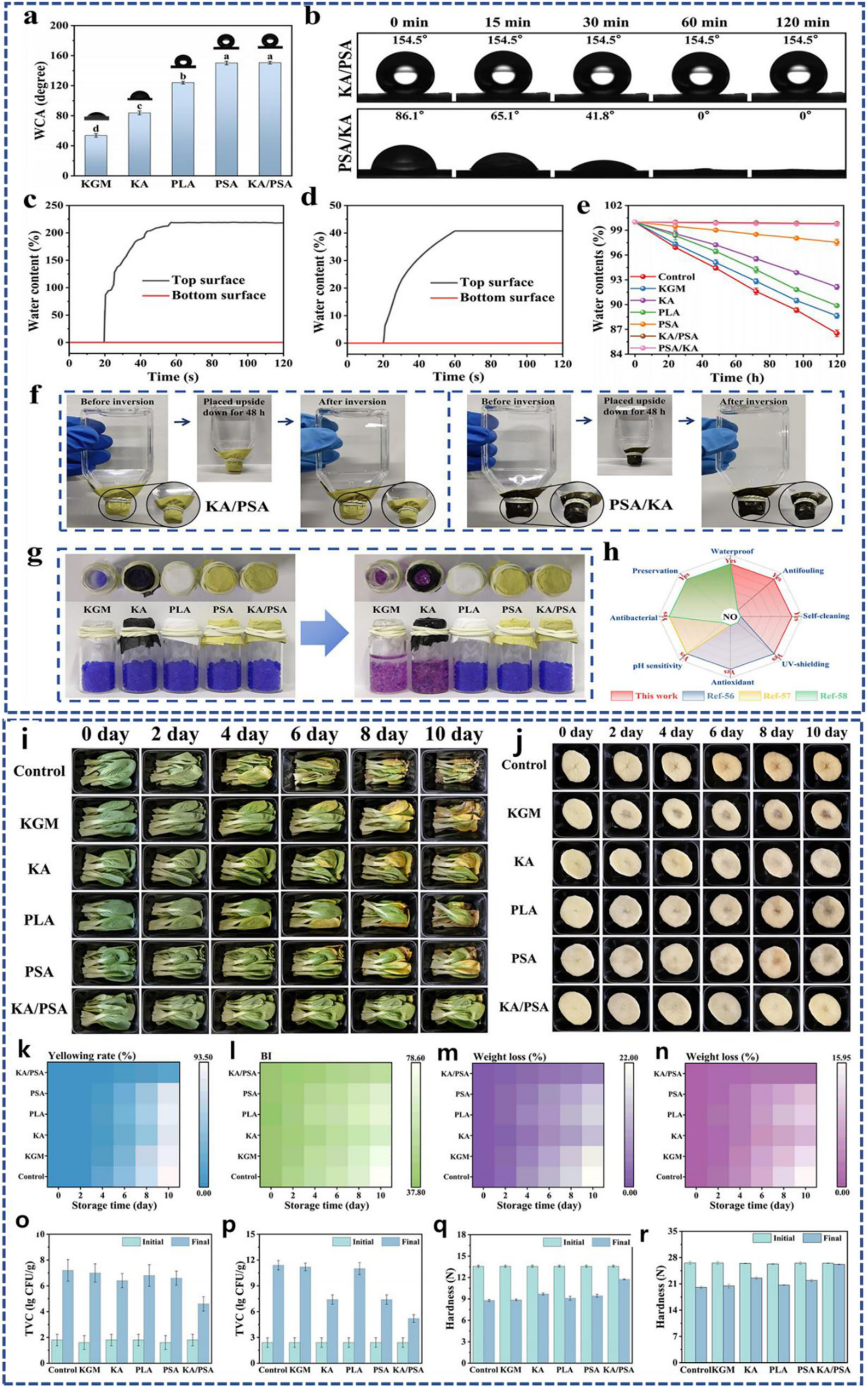
Performance of electrospun fibers in waterproof and food‐preserving packaging applications: (a and b) Water contact angles (WCA) and their stability over time; (c and d) moisture migration results as a function of fiber layer orientation; (e) water vapor transmission rate and (f) sealing integrity; (g) visual seal verification using silicone discoloration. (h) Comparative multifunctionality analysis of KA/PSA electrospun fibers versus existing Janus films. Food preservation results: (i–j) visual appearance of stored pakchoi and potatoes; (k–r) quantitative evaluations of yellowing, browning index (BI), weight loss, total viable count (TVC), and hardness. Reprinted from (Wu, Wang, et al. 2025). KGM, konjac glucomannan; PLA, polylactic acid; WCA, water contact angle.

Bioinspired hydrophobic films mitigate surface moisture and microbial contamination on fresh food, thereby extending freshness. A notable example, inspired by the lotus leaf, combines konjac glucomannan, PLA, and tea polyphenols to form a fiber‐like film designated KA/PSA, exhibiting a WCA >150° and self‐cleaning properties. KA/PSA electrospun fibers exhibit excellent waterproofing, self‐cleaning, and hermetic sealing due to their superhydrophobic surface, making them highly effective for moisture‐resistant, leak‐proof food packaging applications (Figure 11a–h). KA/PSA electrospun fibers successfully maintained the freshness of cut pakchoi and potatoes by delaying browning, weight loss, microbial proliferation, and deterioration in firmness (Figure 11i–r). Their potent antioxidative, antibacterial, and moisture‐retention attributes considerably prolonged shelf life and preserved visual and textural integrity throughout storage. When used for packaging vegetables, it extended the shelf life of cabbage to around 6 days and potatoes to around 10 days, compared to significantly shorter durability without such packaging (Wu, Wang, et al. 2025). A separate work was inspired by the distinctive hydrophobic but adhesive surface of rose petals, using an SNFs modified with hydrophobic ATA. The rose petal‐inspired SNFs/ATA film exhibited a WCA of approximately 134° and successfully preserved cherry tomatoes for 15 days, compared to around 8 days in the control samples (Xie, Feng, et al. 2024). The coating significantly inhibited tomato degradation by repelling water droplets and preventing mold proliferation. Grapes have also profited: a lotus‐inspired film composed of soybean polysaccharide and carnauba wax (a botanical wax) attained a superhydrophobic WCA of around 157° (Z. Ma et al. 2023). Grapes encased in this film remained consumable and untainted for 7 days, although unwrapped grapes often deteriorate considerably more rapidly. In addition, Y. Xie et al. (2026) recently created a hydrophobic Janus starch film with citral nanoemulsions and myristic acid, resulting in a WCA of 118.22°. This dense outer layer decreased moisture penetration by 62.39%, improved durability, and maintained the freshness of goods, illustrating the significance of hydrophobic surface engineering in biodegradable food packaging. Overall, for fresh produce susceptible to microbial spoilage, biopolymer‐based superhydrophobic coatings help maintain an optimal moisture balance by minimizing surface wetness while limiting excessive dehydration. This moisture regulation can ultimately contribute to an extended postharvest shelf life.

Anthocyanin (ATH)‐curcumin (CUR)‐tannic acid (TA)‐based nanoparticles designated as ACT NPs; konjac glucomannan (KGM)/tea polyphenols (TP)/ACT NPs denoted as KA; stearic acid (SA)‐ACT NPs; polylactic acid (PLA)/SA‐ACT NPs designated as PSA.

#### 7.1.3 Bakery Products

Baked foods and snacks pose distinct moisture challenges. They often require protection from humidity to preserve texture and, in some cases, from moisture loss to prevent staling. Crispy items such as chips, crackers, or cookies quickly lose their desirable crunch upon absorbing moisture from the environment (Hu et al. 2025). Biopolymer films with bioinspired hydrophobic surfaces, such as coatings that mimic insect cuticle wax or lotus leaves, can provide an effective moisture barrier to maintain product dryness. Although research specifically addressing superhydrophobic packaging for bakery products is rare, the primary advantage is clear. Hydrophobic, sustainable packaging will reduce humidity infiltration, thereby maintaining the crispness and quality of dry snacks throughout storage. In a report, Hosen et al. produced biodegradable, water‐resistant papers from agricultural waste banana plant (BP) and water hyacinth (WH), coated with ethyl cellulose (EC) (∼10 µm). The EC layer reduced moisture content (6%–11%) and vapor transmission (22%–30%), while improving tensile strength (13%–35%) and preserving surface dryness for over 20 min (Hosen et al. 2022). Coated papers provided UV protection and enhanced transparency within the visible spectrum. This eco‐friendly packaging solution might be considered a viable candidate to replace single‐use plastics in packaging.

Moreover, for bread and other high‐moisture‐sensitive baked products, controlling mold growth and staling is essential. In these products, active packaging complements moisture control. Edible films made from polymers such as starch or proteins, enhanced with natural antimicrobials, have been shown to extend the mold‐free shelf life of bread (Hu et al. 2025). A recent study developed a biodegradable film that incorporates slow‐release clove essential oil, an effective antifungal agent. This dynamic coating served as a moisture barrier while consistently emitting a low concentration of clove oil vapors. The shelf life of bread was extended to 9 days, whereas unprotected bread showed visible mold within a few days, resulting in about a 130% increase in shelf life (Chang et al. 2025). The film was composed of edible biopolymers and degraded by about 70% over 20 days, indicating its suitability for sustainable bakery packaging. Another study on bakery packaging reports that Lopes et al. (2014) produced cinnamaldehyde (CIN)‐infused antimicrobial films for preservative‐free bread and pastries. These films reduced microbial growth by up to four logs in bread and three logs in pastry over 12 and 60 days, respectively. The pastry's hydrophobic fat content enhanced CIN migration, improving antimicrobial action. Despite higher diffusion, sensory impact was minimal. The films’ moisture‐independent activity offers a promising hydrophobic solution for shelf life extension. Gonon et al. (2023) designed PLA‐based thermoformed trays impregnated with carvacrol (CAR) and CIN to improve antibacterial efficacy and water resistance for bakery packaging. The addition of essential oils enhanced the hydrophobicity of PLA by creating a convoluted pathway for water molecules, thereby decreasing vapor permeability. Although elevated EO concentrations somewhat reduced mechanical strength, they significantly inhibited mold proliferation. PLA trays with 5% CIN provided the optimal balance between mold inhibition and dimensional integrity, prolonging cake shelf life from 2 to 3 days. At 8% EO, mold incidence was further reduced, affirming the viability of EO‐loaded PLA as sustainable active packaging. Therefore, moisture‐resistant active films help preserve the textural and microbial quality of bakery products by preventing moisture‐induced softening of dry foods. When incorporated with natural antimicrobials, these films can also inhibit mold growth in moist baked goods, thereby extending shelf life and reducing food waste.

#### 7.1.4 Dairy and Liquid Foods

**FIGURE 12:**
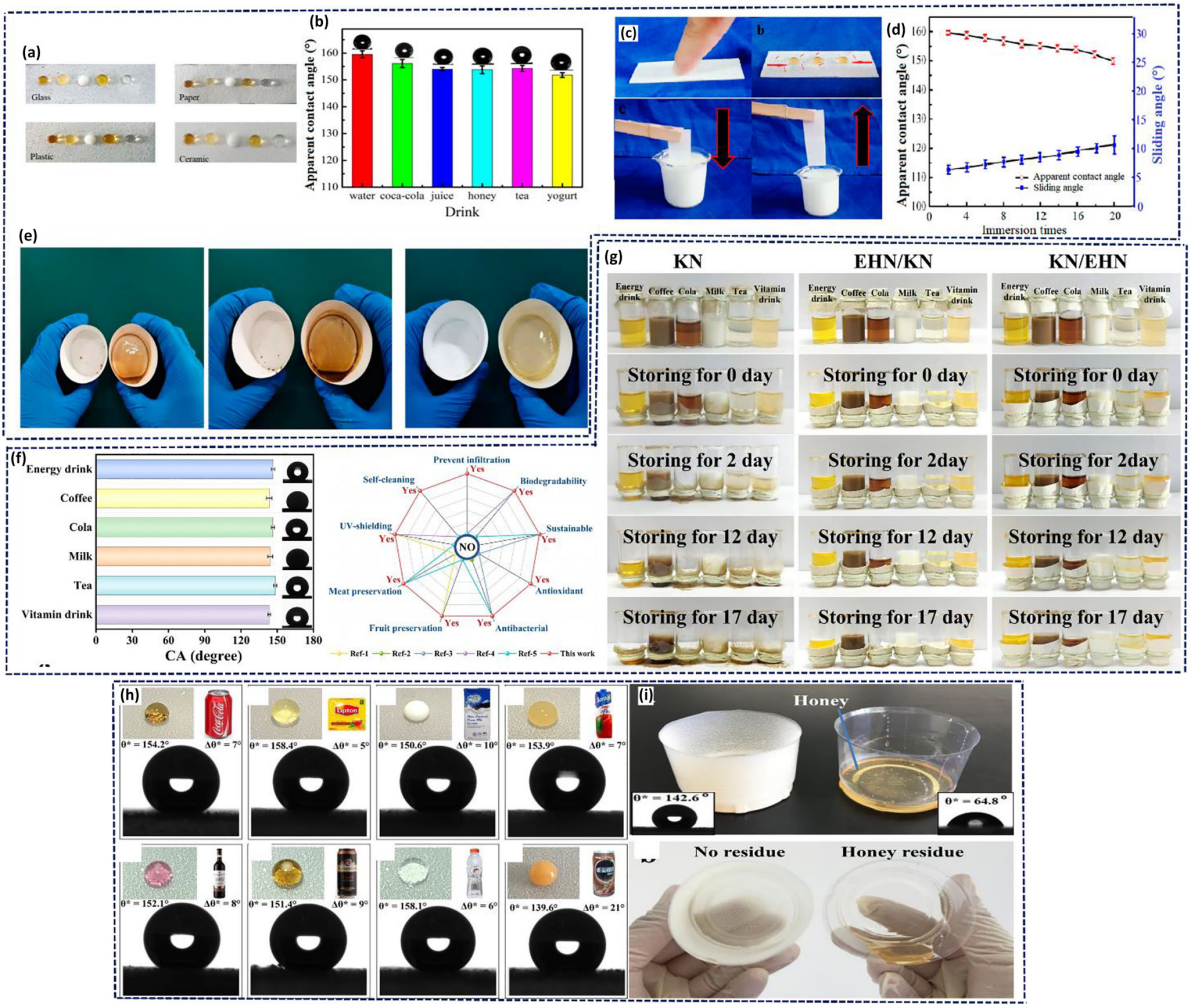
Demonstrations of superhydrophobic coatings on various surfaces and their performance with liquid foods: (a) images showing droplets of Coca‐Cola, black tea, yogurt, honey, and water on different coated substrates; (b) apparent contact and sliding angles on coated glass. (c) durability tests after fingertip pressing and yogurt immersion, followed by droplet behavior post‐interaction. (d) Changes in contact and sliding angles after repeated yogurt immersion. (e) Comparison of residual milky tea, chocolate syrup, and honey in soybean wax‐coated versus uncoated cups (Shen et al. 2020). (f) Contact angle of konjac glucomannan and ethylcellulose (KN/EHN) electrospun fibers and (g) their liquid infiltration resistance versus other Janus membranes for food packaging (Z. Wu et al. 2024). (h) Water contact angles of edible superhydrophobic films with commercial beverages (Coca‐Cola, green tea, milk, juice, wine, beer, Gatorade, and coffee). (i) Liquid retention in uncoated versus coated cups after honey is poured (Y. Li et al. 2018).

Developing biodegradable packaging for liquid or high‐moisture foods (such as milk, dairy beverages, and sauces) has proven challenging because of the need for excellent water resistance. Bioinspired methodologies are enabling the development of biodegradable cartons, cups, and pouches that can contain liquids without leakage or dissolution (Ahuja et al. 2024). For instance, Shen et al. (2020) created an edible, bioinspired superhydrophobic coating using soybean wax, which was applied by a straightforward spray technique. Figure 12a shows that droplets maintained a spherical morphology on coated substrates, indicating non‐wettability. The coated surfaces demonstrated remarkable repellency to various viscous liquid foods, including water, Coca‐Cola, tea, honey, and yogurt, with contact angles between 152° and 159° (Figure 12b). The coating retained exceptional water repellency despite contact with fingers, abrasion, immersion in hot water, and exposure to yogurt (Figure 12c,d). In practical tests, paper cups coated with soybean wax released sticky liquids like honey and chocolate syrup with almost no residue, unlike uncoated cups (Figure 12e), confirming their durability and effectiveness for liquid food packaging.

In another report, Z. Wu et al. (2024) developed bioinspired Janus electrospun fibers for liquid food packaging, combining a hydrophilic konjac glucomannan inner layer with a hydrophobic ethylcellulose outer layer, abbreviated as KN/EHN. The inner layer incorporated *ε*‐poly‐l‐lysine and tannic acid‐ferric ion‐coated CNCs (PTA@CNC NPs) to mimic mussel adhesion. Inspired by lotus leaves, mussels, and red blood cells, the KN/EHN fibers achieved superhydrophobicity (*θ* > 150°) and blocked liquid infiltration for 17 days. KN/EHN electrospun fibers showed strong, stable hydrophobicity (*θ* > 143°) against various liquid foods, including milk, tea, coffee, cola, energy drinks, and vitamin drinks (Figure 12f,g). They prevented leakage for up to 17 days, outperforming conventional Janus membranes and offering a durable, sustainable solution for advanced liquid food packaging.

Furthermore, Y. Li et al. (2018) designed a bio‐inspired, edible superhydrophobic surface using honeycomb wax, Arabic gum, and gelatin, mimicking the pattern of lotus leaves. This multiscale surface achieved high contact angles (*θ* > 150°) for most liquid foods (Figure 12h), indicating superior water repellency and reduced residue. Only coffee showed reduced efficiency (*θ* = 139.6°), presumably due to emulsifiers altering surface interactions. To enhance durability, a flexible, edible elastic film was layered beneath the interface, maintaining superhydrophobicity even after repeated folding and rinsing. Practical testing with polystyrene cups filled with viscous honey (Figure 12i) demonstrated that the interface facilitated residue‐free pouring, thereby validating its efficacy for liquid food packaging.

Such a coating could potentially replace the plastic or wax linings in paper cups, milk cartons, or yogurt tubs, enabling fully biodegradable, waterproof containers for liquids. Although these applications are still in development, early results are promising. The moisture control provided by bioinspired packaging for liquids ensures that dairy foods maintain quality (preventing excessive dehydration or contamination) and that the package remains intact until intended opening. With ongoing advances, consumers may soon see biodegradable pouches or bottles for liquids that rival the performance of today's plastic containers in keeping foods fresh and uncontaminated.

Overall, the available durability studies indicate promising short‐term resistance of biomimetic hydrophobic surfaces to wetting, folding, pressing, and limited mechanical stress. However, these tests do not fully reproduce the cumulative effects of prolonged humidity fluctuations, repeated wetting/drying cycles, flexing, rubbing, stacking pressure, and abrasion encountered during industrial processing, storage, transportation, and handling. Therefore, the long‐term stability of these superhydrophobic surfaces under realistic packaging conditions remains insufficiently established. Future studies should adopt standardized durability assessments, including cyclic humidity exposure, repeated wetting/drying, abrasion, and flex/fold testing. These evaluations are essential to determine whether the initial water‐repellent properties can be retained throughout the intended packaging lifetime.

### 7.2 Benchmarking Biomimetic Films Against Commercial Plastics

Commercial polyolefins and polyester remain the industry benchmark for moisture‐barrier performance. Typical WVTR values vary among conventional packaging polymers: approximately 10–30 g/m^2^/day for low‐density polyethylene (LDPE), 9–11 g/m^2^/day for cast polypropylene (PP), and 2–6 g/m^2^/day for biaxially oriented polypropylene (BOPP). Polyethylene terephthalate (PET) generally exhibits WVTR values of approximately 10–25 g/m^2^/day, depending on film orientation, coating, thickness, and testing conditions (BOPP vs. PET Film 2025; POLFILM 2025; Poly Print. n.d. 2026). PET additionally provides a strong oxygen barrier, with an oxygen transmission rate (OTR) of roughly 10–130 cm^3^/m^2^/day, and considerably higher tensile strength (55–250 MPa) than PP (BOPP vs. PET Film 2025; Nisticò 2020). However, high‐density polyethylene (HDPE) typically shows tensile strength in the range of 20–37 MPa (Industrial Plastic Materials, HDPE n.d.).

Among the biomimetic films discussed in this review, several approaches, or, in specific metrics, exceed these commercial benchmarks. The STA ‐modified CFS (CFS‐30), inspired by the hydrophobic surface of sugarcane peel, achieved a WCA of approximately 153° and a tensile strength of 67.15 MPa. This strength exceeded typical HDPE values and approached those of PET, although its WVTR was compared only with paper straws rather than PE, PP, or PET (Qin et al. 2022). The duck‐feather‐inspired CMC/gelatin film coated with candelilla wax reached a WCA of approximately 142° and extended the refrigerated shelf life of beef to about 5 days, compared with roughly 3 days for conventional PE wrap, indicating a functional barrier improvement even though its WVTR was not directly quantified against PE (Wang, Jiang, et al. 2025). The Janus electrospun konjac glucomannan/ethylcellulose fiber membrane blocked liquid infiltration for 17 days and sustained a WCA between approximately 143° and 150° across various beverages, demonstrating durability under conditions relevant to liquid food packaging, although a direct WVTR comparison with PET was not reported (Z. Wu et al. 2024). The nacre‐inspired montmorillonite/graphene nanocomposite film reduced WVTR by roughly an order of magnitude relative to the unmodified biopolymer matrix, moving its barrier performance closer to, though still generally below, the PP‐to‐PET range (H. Zhao et al. 2018). Similarly, the rice‐bran‐wax and chitosan–curcumin coating increased WCA from about ∼84° to ∼130° while lowering wvp, again illustrating meaningful but partial progress toward commercial‐plastic‐level performance (T. Liu et al. 2025).

Taken together, these comparisons show that select biomimetic films, particularly nanocellulose‐ and wax‐modified systems, can match or exceed PE and PP in WCA and, in some cases, tensile strength. However, most biomimetic films still fall short of PET's combined mechanical strength and oxygen‐barrier performance, and few studies report WVTR under standardized testing conditions directly comparable to those used for commercial plastics. This inconsistency in testing protocols, rather than an inherent performance limitation, remains a major obstacle to rigorous one‐to‐one comparisons. Standardized side‐by‐side testing is therefore essential before biomimetic films can be reliably considered direct alternatives to PE, PP, or PET for demanding food packaging applications.

## 8 Challenges and Prospects for Bioinspired Food Packaging Materials Design

Despite significant advances in bioinspired, water‐resistant biopolymer packaging, several critical challenges remain before these materials can achieve widespread commercial use. Historically, many reviews and innovations have emphasized other functional attributes (e.g., mechanical strength, active packaging features) over water resistance, even though moisture sensitivity is a primary weakness of biopolymer films. Moving forward, a concerted focus on improving water barrier performance is essential. Below, we discuss key hurdles and emerging strategies for scalable implementation, including ensuring biodegradability, leveraging advanced design tools, and aligning with sustainability and circular economy goals.

### 8.1 Scalability and Reproducibility

A major challenge is scaling up laboratory results for bioinspired hydrophobic coatings and nanocomposites to industrial‐scale manufacturing. Numerous methods used to fabricate superhydrophobic or water‐repellent surfaces (e.g., lithography, chemical vapor deposition, and electrospinning) are efficient at the small scale but are too complex or costly for large‐scale production (Acha et al. 2025). These sophisticated manufacturing techniques impede the economical, high‐volume manufacture of packaging films. Achieving uniform micro/nano‐structuring or consistent nanofiller dispersion over large areas can be challenging, resulting in variability in film performance. Ensuring uniformity and repeatability across batches of biopolymer nanocomposites is essential (Koirala et al. 2025).

Replicating surface patterns at an industrial scale remains a major challenge. Hierarchical micro‐ and nanoscale roughness, such as lotus‐like papillae or feather‐barb structures, is generally produced using lithography, plasma etching, or template‐assisted molding on relatively small laboratory areas at the cm^2^ scale. In contrast, industrial techniques such as roll‐to‐roll imprinting and hot embossing must reproduce these structures uniformly across meter‐wide webs while maintaining accurate pattern replication, consistent coating thickness, and defect‐free registration at much higher production speeds than laboratory casting processes (Acha et al. 2025). Cost analysis also highlights this laboratory‐to‐industry gap. For example, the CFS‐30 straw was estimated to cost approximately 0.7 cents per unit, compared with about 2 cents for PLA and 4 cents for paper straws under laboratory‐scale assumptions (Qin et al. 2022). However, similar techno‐economic assessments comparing coated or micro‐patterned biomimetic films with commercial PE, PP, and PET materials (Section 7.2, benchmarking discussion) are still largely unavailable. Consequently, a reliable comparison of their industrial‐scale production costs cannot yet be made.

To address scalability, researchers are investigating simpler, more scalable coating and casting methods. Techniques such as spray coating, dip coating, roll‐to‐roll imprinting, and LBL assembly are being refined for the industrial‐scale production of biomimetic waterproof surfaces (Acha et al. 2025). These methods can more effectively integrate nature‐inspired hierarchical patterns into packaging materials. For instance, low‐cost replication of the lotus‐leaf effect (micro/nano dual roughness) by spray‐coating hydrophobic nanoparticles has been demonstrated as a viable route to create water‐repellent films in a roll‐to‐roll process. Similarly, bioinspired nanocomposites can be produced via conventional extrusion or casting if the formulations are adjusted for processability (Iftekar et al. 2023). Continued engineering is needed to retrofit existing packaging processes or develop new techniques for bio‐nanocomposites, alongside rigorous quality control to ensure reproducible water‐resistant surface textures and uniform filler dispersion at the pilot scale. Another related challenge is the mechanical durability of these bioinspired surfaces. The very micro/nanostructures that confer superhydrophobicity are often fragile and prone to abrasion or wear during processing and use (Acha et al. 2025). Improvements in mechanical robustness will go hand‐in‐hand with scaling up, since industrial handling demands resilience.

A related and largely unresolved issue is the long‐term functional stability of these hierarchical surfaces. Most reported superhydrophobic biopolymer films are evaluated using short‐term wetting, limited folding/rinsing cycles, or brief abrasion tests. In contrast, standardized protocols simulating realistic industrial and retail conditions, such as repeated 24–48 h high‐/low‐humidity cycles, multiple wetting‐drying cycles, and quantitative abrasion testing, are still largely lacking. Because micro‐ and nanoscale surface roughness is the principal contributor to superhydrophobicity, physical wear, plastic deformation, or embedding of contaminants into these features can progressively reduce the WCA and increase contact‐angle hysteresis, thereby compromising water repellency well before the packaging reaches the end of its intended shelf life. Establishing standardized durability metrics and reporting WCA retention (%) after defined humidity‐cycling and abrasion protocols should therefore become a routine requirement for validating the industrial readiness of bioinspired water‐resistant packaging materials.

On the basis of the technology readiness level (TRL) framework, most bioinspired water‐resistant films reviewed here remain at TRL 2–4, meaning they are mainly at the laboratory validation stage (Jain and Kumar 2026). This is consistent with other nanomaterial‐based food packaging systems (Khojastehnezhad et al. 2026) and is reflected by studies limited to small‐scale WCA and WVTR testing under controlled conditions. Similarly, nanocellulose composites have reached about TRL 5 for general applications, whereas their use in food packaging remains at lower TRLs because of additional food contact requirements (Jain and Kumar 2026; Silva et al. 2020). Only a few systems, including roll‐to‐roll spray‐coated lotus‐mimetic films (Acha et al. 2025) and extrusion‐processed bio‐nanocomposites (Iftekar et al. 2023), show higher technological readiness. However, none have yet been demonstrated under real industrial packaging‐line conditions. Therefore, further engineering development, standardized durability testing, and techno‐economic evaluation are needed before commercial application.

### 8.2 Balance of Water Resistance and Biodegradability

Improving the water resistance of a biopolymer film can inadvertently hinder its biodegradability, posing a significant trade‐off. Hydrophobic modifications (such as surface acetylation, wax or silane coatings, or the addition of hydrophobic nanoparticles) tend to slow water ingress, which, in turn, slows microbial colonization and hydrolytic breakdown. For example, silane‐treated seaweed/cellulose films exhibited substantially lower degradation rates in soil burial analysis than unmodified hydrophilic films (Surya et al. 2022). The unmodified films lost ∼75% of their mass in 30 days, whereas the hydrophobic silane‐coated films lost only ∼53%, indicating that water‐repellent films persist longer before biodegrading. These results highlight the challenge: waterproofing a biodegradable polymer often makes it more “plastic‐like” in its resistance to environmental breakdown.

Future research is focusing on intelligent design strategies to resolve this conflict. One approach involves multilayer or hybrid structures that decouple functionality: for example, an outer hydrophobic layer for water resistance and an inner hydrophilic or enzyme‐sensitive layer that ensures eventual disintegration. A notable example is the recently reported LEAFF material (Layered, Ecological, Advanced, and Multifunctional Film) inspired by plant leaves (Dhatt et al. 2025). Dhatt et al. (2025) engineered a multilayer biomimetic composite on the basis of a PLA matrix that demonstrated superior water stability and gas barrier properties, prolonging shelf life, while fully biodegrading in ambient soil after 5 weeks (Dhatt et al. 2025). By mimicking the layered structure of leaves, they enhanced mechanical strength and water resistance while introducing components that initiate fast biodegradation upon disposal. These designs demonstrate that it is possible to achieve a balance between water resistance and biodegradability via meticulous material selection and structural design.

Another strategy is to use fully bio‐based hydrophobic agents (such as plant waxes, fatty acid coatings, or hydrophobic amino acid derivatives) that are inherently biodegradable, albeit at a gradual rate. These can provide a transient moisture barrier during product application, though they ultimately degrade over an extended period. For example, beeswax or carnauba wax coatings on biopolymers enhance water resistance and are biodegradable, but at a slower rate than polysaccharides, as bacteria ultimately decompose them (Kończak et al. 2025). Researchers are also investigating triggerable systems, for example, coatings that maintain integrity during a product's shelf life but may be removed or auto‐degrade under composting conditions, potentially through pH alterations or enzymes released by microbes. Achieving adherence to composability criteria is a future objective for water‐resistant, sustainable packaging. Overall, design for end‐of‐life is becoming as important as design for performance. The next generation of bioinspired packaging must maintain a delicate balance, providing adequate water resistance in use while not persisting indefinitely in the environment.

### 8.3 Food Contact Safety and Regulatory Considerations for Nanomaterial‐Enhanced Films

Beyond the material‐specific concerns, nanomaterial‐enhanced packaging faces three main safety considerations: migration, toxicity, and regulatory authorization. Nanoparticles or their dissolved constituents may migrate from packaging into food, and the extent of migration can depend on particle characteristics, filler concentration, polymer structure, food composition, contact time, and temperature (Zhou et al. 2024). Therefore, migration should be evaluated under conditions that reflect the intended food and storage conditions. In the EU, Regulation (EC) No. 1935/2004 requires food contact materials not to transfer constituents to food at levels that could endanger human health, whereas Regulation (EU) No. 10/2011 specifies appropriate food simulants and migration‐testing conditions for plastic materials (More et al. 2021). Importantly, substances in nanoform may be used in plastic food contact materials only when explicitly authorized for that nanoform under Regulation (EU) No. 10/2011.

Toxicological assessment should also consider nanoscale‐specific properties, including particle size and distribution, surface chemistry, shape, dissolution/degradation behavior, exposure level, and potential accumulation, because safety information for the corresponding bulk material cannot automatically be applied to its nanoform (More et al. 2021). In the United States, FDA evaluates nanotechnology‐containing products within its existing regulatory framework on a product‐ and use‐specific basis (FDA 2014; U.S. Food and Drug Administration 2021).

Food contact substances that require authorization generally follow the Food Contact Notification or other applicable premarket pathways, rather than being considered automatically acceptable because the corresponding conventional material has an established safety status. Therefore, commercial translation of nanomaterial‐enhanced biomimetic packaging requires standardized migration studies, nanospecific toxicological assessment, and compliance with the applicable EU or FDA authorization requirements, in addition to demonstrating improved packaging performance.

### 8.4 Advanced Characterization, Modeling, and Machine‐Learning‐Assisted Design

To accelerate improvements in water‐resistant biopolymer systems, advanced characterization and computational modeling tools are being extensively used. Characterization techniques are evolving to enhance understanding of water's interactions with these complex materials. For instance, advanced microscopy and tomography can elucidate the three‐dimensional distribution of nanofillers and pores, facilitating correlation between microstructure and WVTRs. High‐sensitivity gravimetric and spectroscopic methods enable real‐time monitoring of moisture uptake or diffusion in biopolymer films. Such detailed analyses guide the refinement of compositions and coatings to plug any microscopic pathways for water.

On the modeling front, researchers are using simulations to predict barrier properties and optimize designs before experimental trials. Molecular dynamics and Monte Carlo simulations can investigate the diffusion of water molecules within a polymer‐nanoparticle matrix or the effect of specific surface chemistry on the WCA (Dehghani et al. 2017). Finite element models of multilayer films help elucidate stress, diffusion, and degradation behavior under various conditions (Chen and Juang 2016). Machine learning (ML) has emerged as a powerful tool for navigating the large formulation space of biopolymer composites. Instead of laboriously testing countless combinations of biopolymers, plasticizers, cross‐linkers, and hydrophobic coatings, ML algorithms can be trained on existing data to predict optimal formulations that achieve multiple targets (e.g., low water permeability, high strength, and biodegradability).

Recent studies demonstrate the promise of data‐driven design. Gautam et al. (2025) applied an ML‐driven optimization to a starch‐chitosan edible film containing glycerol, beeswax, ZnO nanoparticles, and citrus extract (Gautam et al. 2025). By training a neural network in conjunction with response‐surface methodologies, they identified a formulation that reduced WVP by 31% and enhanced tensile strength, while remaining entirely soil‐biodegradable within 45 days. This method reduced the experimental search space by 65%, demonstrating how AI may accelerate the creation of high‐performance biodegradable packaging. AI‐driven material design is identified as a growing trend for next‐generation biopolymers (Agbogo et al. 2025). Researchers are increasingly employing generative algorithms to create surface textures (e.g., superhydrophobic patterns) and to identify innovative bio‐based additives that improve water resistance without compromising other properties. In the coming years, the integration of ML with high‐throughput experimentation (e.g., automating the synthesis and testing of film libraries) might significantly accelerate innovation. These methodologies will both profit from and enhance extensive databases of material attributes, a crucial advancement for data‐informed design of sustainable packaging (Agbogo et al. 2025).

### 8.5 Considerations for Sustainability and the Circular Economy

Advancements in water‐resistant biopolymer packaging must align with the principles of green chemistry and the circular economy. This involves using sustainable sourcing and production methods while considering the material's end‐of‐life management. Many bioinspired solutions to date have relied on chemicals or nanomaterials that, while effective, raise environmental or health concerns. For instance, achieving superhydrophobicity in laboratory experiments often uses fluorinated silanes or PFAS‐based coatings, which are persistent pollutants. There is an initiative to eliminate such chemicals from food packaging applications. Researchers emphasize developing fluorine‐free, nontoxic hydrophobic coatings, such as those using biodegradable polymers or naturally sourced hydrophobes, to ensure adherence to environmental and regulatory standards (Acha et al. 2025).

Compliance with ecological regulations is essential; a highly water‐resistant film is economically unviable if it leaches toxins or fails to meet food contact safety requirements. Governments and consumers are becoming more cautious about nanoparticles in food contact, necessitating comprehensive risk studies for any nano‐enhanced packaging (Koirala et al. 2025). Research must evaluate the potential migration of nanoparticles or monomers into food or the environment during use and after disposal, highlighting that uncertainties about nanomaterial cytotoxicity and insufficient migration data pose significant obstacles to adoption. A cautious approach is thus justified, emphasizing GRAS (generally recognized as safe) components and comprehensively understanding the disposition of each component in composting or recycling processes.

From a circular economy perspective, the optimal outcome is that these materials either biodegrade harmlessly or are recycled or reused in a closed loop. Biodegradable films require a compostable polymer matrix and additives (such as nanofillers and coatings) that do not produce pollutants or nondegradable residues. Inorganic nanofillers like nanoclays or silica may be harmless at low concentrations but will remain as mineral particles; therefore, modest application or a transition to biodegradable nanofillers (e.g., nanocellulose, starch nanocrystals) is advisable. Certain studies use agricultural waste as a polymer source and a hydrophobic ingredient, exemplified by films composed of starch/protein extracted from food waste and coated with wax generated from plant‐based oils (Onyeaka et al. 2022), thereby promoting waste valorization and compostability into nontoxic goods.

Compliance with ecological regulations is essential; a highly water‐resistant film is economically unviable if it leaches toxins or fails to meet food contact safety requirements. Governments and consumers are becoming more cautious about nanoparticles in food contact, necessitating comprehensive risk studies for any nano‐enhanced packaging. Research must evaluate the potential migration of nanoparticles or monomers into food or the environment during use and after disposal, highlighting that uncertainties about nanomaterial cytotoxicity and insufficient migration data pose significant obstacles to adoption. A cautious approach is thus justified, emphasizing GRAS components and comprehensively understanding the disposition of each component in composting or recycling processes.

From a circular economy perspective, the optimal outcome is that these materials either biodegrade harmlessly or are recycled or reused in a closed loop. Biodegradable films require a compostable polymer matrix and additives (such as nanofillers and coatings) that do not produce pollutants or non‐degradable residues. Inorganic nanofillers like nanoclays or silica may be harmless at low concentrations but will remain as mineral particles; therefore, modest application or a transition to biodegradable nanofillers (e.g., nanocellulose, starch nanocrystals) is advisable. Certain studies use agricultural waste as a polymer source and a hydrophobic ingredient, exemplified by films composed of starch/protein extracted from food waste and coated with wax generated from plant‐based oils, thereby promoting waste valorization and compostability into nontoxic goods.

Process sustainability is also significant: pathways that require elevated temperatures, harsh solvents, or many steps increase the environmental footprint. Future prospects include solvent‐free processing, the use of green solvents (such as water and ethanol), and bio‐catalytic modifications (for instance, enzyme‐catalyzed cross‐linking or grafting) to replace harsh chemical methods. Life‐cycle assessment (LCA) is essential to validate actual benefits, and several studies indicate that, despite more processing, biopolymer packaging may still outperform petrochemical plastics in total environmental footprint and end‐of‐life effects (Dhatt et al. 2025). Full life‐cycle LCA studies comparing biopolymer films with conventional plastics commonly evaluate environmental impacts such as global warming potential, fossil‐resource depletion, and eutrophication. These studies show that adding nanofillers or applying additional coating steps can change the overall environmental impact, highlighting the importance of conducting LCA alongside barrier performance testing rather than using it only after material development to justify sustainability (Spierling et al. 2018).

### 8.6 Research Roadmap for Next‐Generation Bioinspired Packaging

Future research should move beyond improving individual film properties and toward an integrated design validation manufacturing pathway. In the near term, AI and ML should progress from simple formulation screening toward performance‐driven design, where desired properties such as WCA, WVP, WVTR, mechanical strength, biodegradability, food contact safety, and cost are defined first, and suitable materials and structures are then predicted. The feasibility of this approach is supported by ML‐assisted optimization of bio‐based packaging formulations and bioinspired brick‐and‐mortar composites (Gautam et al. 2025; Morsali et al. 2020). A major priority is therefore to establish standardized datasets that include not only initial film properties but also performance after humidity cycling, abrasion, repeated wetting/drying, migration testing, and degradation. This would allow AI models to optimize practical packaging performance rather than laboratory properties alone.

A second emerging direction is digital‐twin‐assisted packaging development. Digital twins combine experimental measurements with computational models to create virtual representations of packaging materials that can be tested under different conditions. Zarei et al. (2024) demonstrated this approach for paper‐aluminum packaging laminates by validating a finite‐element model experimentally and using it to predict mechanical behavior (Zarei et al. 2024). For biomimetic biopolymer films, future digital twins could integrate surface roughness, coating thickness, WCA, moisture diffusion, temperature, relative humidity, and mechanical stress to predict when hydrophobic or barrier performance begins to deteriorate. Coupling such models with AI/ML could reduce trial‐and‐error experiments and support formulation and process optimization before pilot‐scale production.

Another priority is multifunctional moisture‐responsive packaging. Instead of providing a fixed barrier throughout storage, future materials could respond to changes in package humidity by adjusting moisture transport or releasing active compounds only when required. Humidity‐ and pH‐responsive antimicrobial nanofibers have already demonstrated environmentally triggered release of active agents (Jiao et al. 2024), whereas moisture‐sensitive alginate/carboxymethyl‐cellulose films provide another approach for responsive antimicrobial packaging (Tan et al. 2024). These concepts could be combined with biomimetic hydrophobic surfaces to simultaneously control moisture, inhibit microbial growth, and monitor food deterioration. However, response thresholds should be tailored to specific foods, and repeated‐response stability and migration safety must be validated.

Self‐healing hydrophobic coatings offer a complementary strategy for improving durability (Celik et al. 2023). As reversible self‐healing chemistry is already established, the next challenge is to ensure that repair restores not only mechanical integrity but also surface hydrophobicity and moisture‐barrier performance after scratching, folding, or abrasion. Self‐healing superhydrophobic coatings have demonstrated recovery of water repellency after surface damage (Celik et al. 2023). Future packaging studies should therefore measure the recovery of WCA, WVP, WVTR, seal integrity, and mechanical properties over repeated damage‐healing cycles under realistic storage conditions.

Finally, 3D printing offers a promising route for precisely constructing hierarchical biomimetic structures that are difficult to control using conventional casting or coating. High‐resolution printing has already produced non‐fluorinated lotus‐inspired structures with tunable superhydrophobicity (Trink and Magdassi 2024). For packaging, this approach could enable controlled microtextures, multilayer barriers, directional moisture pathways, and locally reinforced structures. Key priorities are developing food‐safe printable biopolymers, increasing printing speed and resolution, and evaluating scalability. Overall, future development should follow a closed‐loop pathway integrating AI/ML‐guided design, digital‐twin prediction, responsive and self‐healing functionality, precision manufacturing, and standardized validation of food storage performance, safety, durability, and scalability.

## 9 Conclusion

Replacing petroleum‐based plastics with sustainable food packaging has become a global imperative driven by environmental concerns, safety regulations, and consumer demands. Although biopolymers offer promising advantages in terms of biodegradability and food compatibility, their inherent hydrophilicity, coupled with limited water resistance and degraded mechanical properties and barrier properties in wet or humid environments, remains major obstacle to commercial‐scale application.

In this context, this article comprehensively addresses this critical issue by exploring biomimetic design strategies as a powerful tool for enhancing the water‐resistant performance of biopolymers. Drawing on insights gained from examples of natural organisms exhibiting exceptional hydrophobicity and multifunctionality, such as lotus, mussel, feather, nacre, and insect cuticles, this article integrates recent advances in nanocomposite engineering, hierarchical surface structuring, and interfacial chemistry, tailored to food packaging and safety. Through a systematic evaluation of fabrication techniques (e.g., solution casting, electrospinning, and LBL assembly), surface functionalization methods (e.g., plasma treatment, conjugation, and biomimetic coating), and nanomaterial integration, this article presents a framework for designing biomimetic biopolymer films with enhanced hydrophobicity, mechanical strength, and environmental safety. These strategies are presented not as individual technological achievements, but as an interconnected approach that reflects the broader potential of biomimetic materials science in addressing real‐world packaging challenges.

Furthermore, this review highlights the practical applicability of these systems to protect a wide range of foods, including fruits, vegetables, bakery goods, meats, dairy products, and liquids, by improving moisture management and extending shelf life. Furthermore, it presents new challenges, such as improving water resistance while maintaining biodegradability, achieving industrial scale‐up, and ensuring reproducibility of surface properties and nanomaterial dispersions. These challenges represent key areas where future research should be combined with practical engineering. Focusing on water resistance, a relatively under‐researched yet critically important characteristic in food packaging, this review fills a gap in the existing literature and provides useful guidance to researchers and developers. Ultimately, it offers practical insights for those seeking to design next‐generation, eco‐friendly, functional, and scalable packaging systems that can replace synthetic plastics.

Overall, biomimetic and biopolymer‐based waterproof packaging is not simply an academic discipline but a crucial area for innovation. With environmental concerns and increasing plastic regulations worldwide, the approaches discussed in this review provide a roadmap for materials scientists, food technologists, and packaging engineers to collaborate to develop solutions that are not only sustainable but also effective and immediately applicable to industrial settings.

## Author Contributions

**Ajahar Khan**: conceptualization, investigation, methodology, validation, writing – review and editing, writing – original draft, visualization, resources, data curation. **Zohreh Riahi**: investigation, writing – original draft, writing – review and editing, visualization, validation, methodology, data curation, resources. **Ruchir Priyadarshi**: visualization. **Jong‐Whan Rhim**: conceptualization, investigation, funding acquisition, writing – original draft, visualization, validation, writing – review and editing, project administration, supervision.

## Conflicts of Interest

The authors declare no conflicts of interest.

## Data Availability

No new data were produced during this work.
